# Predictive blood biomarkers of sheep pregnancy and litter size

**DOI:** 10.1038/s41598-022-14141-w

**Published:** 2022-06-20

**Authors:** Seyed Ali Goldansaz, Susan Markus, Graham Plastow, David S. Wishart

**Affiliations:** 1 Department of Agriculture, Food and Nutritional Sciences, University of Alberta, Edmonton, T6G 2P5 Canada; 2 Department of Biological Sciences, University of Alberta, Edmonton, T6G 2E9 Canada; 3 Technology Access Centre for Livestock Production, Olds College Centre for Innovation, Olds College, Olds, AB T4H 1R6 Canada; 4 Lakeland College, 5707 College Drive, Vermillion, AB T9X 1K5 Canada; 5 Department of Computing Science, University of Alberta, Edmonton, T6G 2E8 Canada; 6 Department of Laboratory Medicine and Pathology, University of Alberta, Edmonton, AB T6G 2R3 Canada; 7 Faculty of Pharmacy and Pharmaceutical Sciences, University of Alberta, Edmonton, AB T6G 2H7 Canada

**Keywords:** Animal physiology, Diagnostic markers

## Abstract

Early detection of sheep pregnancy and the prediction of how many lambs a pregnant ewe delivers affects sheep farmers in a number of ways, most notably with regard to feed management, lambing rate, and sheep/lamb health. The standard practice for direct detection of sheep pregnancy and litter size (PLS) is ultrasonography. However, this approach has a number of limitations. Indirect measurement of PLS using blood biomarkers could offer a simpler, faster and earlier route to PLS detection. Therefore, we undertook a large-scale metabolomics study to identify and validate predictive serum biomarkers of sheep PLS. We conducted a longitudinal experiment that analyzed 131 serum samples over five timepoints (from seven days pre-conception to 70 days post-conception) from six commercial flocks in Alberta and Ontario, Canada. Using LC–MS/MS and NMR, we identified and quantified 107 metabolites in each sample. We also identified three panels of serum metabolite biomarkers that can predict ewe PLS as early as 50 days after breeding. These biomarkers were then validated in separate flocks consisting of 243 animals yielding areas-under-the-receiver-operating-characteristic-curve (AU-ROC) of 0.81–0.93. The identified biomarkers could lead to the development of a simple, low-cost blood test to measure PLS at an early stage of pregnancy, which could help optimize reproductive management on sheep farms.

## Introduction

Sheep are relatively prolific small ruminants and an important source of animal protein contributing to human diets worldwide. Sheep gestation is relatively short (about 150 days) and litter sizes consisting of two or more offspring are common. As a result, sheep farm profitability is highly correlated to reproductive efficiency. Formally, reproductive efficiency for sheep farmers is expressed as the number of lambs born annually per ewe exposed to a ram at breeding. Breed type and prolificacy, nutrition, environment, age at first mating, conception rate, embryo and fetus viability, and flock age structure are some of the determining factors contributing to reproductive efficiency. However, outcomes of ewe fertility management can vary considerably among flocks. Identifying pregnant ewes and determining the number of fetuses they carry are key components of breeding management in sheep production^1^. Pregnancy testing during the critical early period of the mating season allows for re-breeding or the culling of non-pregnant ewes, resulting in increasing flock pregnancy rates^2^. If producers miss this opportunity, they can adjust their management practices by separating the open ewes from the pregnant mob to feed each group based on their physiological needs. Another benefit to early determination of pregnancy and litter size (PLS) is the acquisition of valuable data for selection and breeding purposes.

In addition to detecting pregnancy, predicting or determining litter size is instrumental to successful reproductive management. Maternal nutrition during gestation directly impacts ewe prolificacy^3^ as well as lamb survivability and performance. These lamb performance traits include growth^4,5^, reproductive capacity^6^ and hormonal development^7^. Thus, early detection of ewe PLS elevates income for producers by increasing the number of pregnant ewes and the number of healthy lambs born. Costs of production are reduced by preventing over-feeding of open ewes, and optimizing rations based on nutritional needs of the pregnant animals in an attempt to reduce the number of overweight singles, small-sized multiples and the incidence of pregnancy toxemia.

Ultrasonography is the gold standard and the most commonly performed method for PLS detection in sheep^8^. This method requires producers to either invest in an ultrasound machine and develop the appropriate skills for scanning or they must contract the services from a veterinarian. Ultrasound pregnancy detection is commonly practiced between 45 and 90 days into gestation^9^. However, detecting the number of fetuses is not straightforward and depends on the time of scanning as well as operator experience^10^. The breeding season is also a busy time for ultrasound professionals, limiting the number of farms they can serve. The cost of ultrasonography, currently CAD$5–8/ewe in Alberta in Canada, also varies depending on flock size and geographical location of the farm. This makes ultrasonography more expensive for medium-to-small size flocks and those that are not conveniently accessible. In some jurisdictions, including the province of Alberta, delivering ultrasound services specifically for pregnancy diagnosis is restricted to veterinarian professionals, which limits its widespread use.

Molecular biomarkers, such as proteins or metabolites found in blood, urine or milk, are a promising alternative for the indirect measurement or prediction of different traits in many livestock species^11,12^. Biomarkers are most suited for traits that have higher economic value. Likewise, biomarkers are particularly useful if the trait measurement needs to be performed within a short timeframe, or if the direct measurement of the trait involves lengthy trials, is labour-intensive, leads to loss of the animal or is expensive. While plasma progesterone (P4) levels can be used to detect sheep pregnancy as early as 18 days, P4 does not accurately detect open, non-pregnant ewes^13,14^. Likewise, there is no commercial kit that provides the service to farmers in any part of the world (including in Alberta). Recent literature indicates promising results when applying metabolomics to detect pregnancy in other livestock species^11,15,16^. However, there are no publications using high throughput metabolomics platforms to characterize non-hormonal metabolite biomarkers that can be used for sheep PLS detection in readily accessible biofluids at early stages of gestation. Therefore, a metabolomic study on early-stage sheep PLS detection is warranted.

Livestock metabolomics is an emerging field that has led to the discovery of useful biomarkers in many livestock species^12^. However, only one study has used metabolomics to investigate non-hormonal metabolic changes during ewe pregnancy^17^. Most other metabolomic studies have measured hormones or individual metabolites associated with ewe pregnancy^18–22^. Previously, we have shown that metabolomics can be used to identify candidate blood biomarkers for detecting several economically important traits in sheep, such as residual feed intake and carcass merit^23^. Based on that success, we decided to investigate if blood biomarkers of sheep PLS could be identified and validated.

Given the metabolic changes that occur due to pregnancy, we hypothesized that ewe pregnancy and the number of lambs delivered per pregnant ewe can be predicted at early stages of pregnancy using blood biomarkers. Therefore, the objectives of this study were to: (1) profile the blood metabolome associated with ewe PLS, and (2) identify and validate blood biomarkers of ewe PLS prior to 60 days of gestation. These findings could provide an alternative route for ewe pregnancy detection and enhance the reproductive management of sheep flocks. Indirect measurement of sheep PLS through blood biomarkers is also expected to increase the profitability of sheep production by reducing the proportion of open ewes during the breeding season. It will also improve the health and welfare of pregnant ewes through better nutritional management based on their pregnancy requirements.

## Results

The results from our metabolomic studies on sheep PLS are divided into three sections. The first describes the changes detected in serum metabolite levels of ewes during different timepoints of pregnancy. The second (discovery phase) describes the identification of serum-based PLS biomarkers at different stages of pregnancy through pairwise comparisons of pregnant and non-pregnant ewes, as well as via pairwise comparisons of pregnant ewes with different litter sizes (based on pregnancy outcome). The third describes validation or replication of the PLS biomarkers identified at day 50 of gestation in the discovery phase on an independent (hold-out) larger cohort of ewes.

### Changes in the serum metabolome of ewes during pregnancy

The first objective of this study was to comprehensively and quantitatively characterize the serum metabolome of ewes from seven days pre-breeding to 70 days post-breeding. The Livestock Metabolome Database (LMDB^12^) currently includes 375 compounds assigned to the sheep metabolome, 300 of which were previously reported and quantified in the serum/plasma metabolome of non-pregnant sheep. As there are no published reports regarding the serum metabolome of sheep during gestation we undertook a targeted, quantitative metabolomic analysis of sheep serum using two analytical platforms, NMR spectroscopy and LC–MS/MS. We were able to identify and quantify 107 metabolites with unique chemical structures in the serum of 131 pregnant/non-pregnant ewes over 5 different timepoints (the classification of these metabolites based on each platform is provided in Table 1). Details regarding the most significant longitudinal changes and most differentiating metabolites are described below.

**Table 1:** Serum metabolome associated with sheep pregnancy.

Platform	Metabolite	LMDB ID	ClassyFire chemical classification
NMR	1-Methylhistidine	LMDB00001	Carboxylic acids and derivatives
	2-Hydroxybutyric acid	LMDB00003	Hydroxy acids and derivatives
	2-Hydroxyisovalerate	LMDB01096	Fatty Acyl derivatives
	3-Hydroxybutyric acid	LMDB00144	Hydroxy acids and derivatives
	3-Hydroxyisovaleric acid	LMDB00238	Fatty Acyl derivatives
	3-Methyl-2-oxovaleric acid	LMDB01097	Keto acids and derivatives
	Acetic acid	LMDB00014	Carboxylic acids and derivatives
	Acetoacetate	LMDB00026	Keto acids and derivatives
	Acetone	LMDB00352	Organooxygen compounds
	l-Arginine	LMDB00171	Carboxylic acids and derivatives
	l-Asparagine	LMDB00075	Carboxylic acids and derivatives
	Betaine	LMDB00015	Carboxylic acids and derivatives
	Butyrate	LMDB00013	Fatty Acyl derivatives
	Choline	LMDB00041	Organonitrogen compounds
	Citric acid	LMDB00040	Carboxylic acids and derivatives
	Creatine	LMDB00029	Carboxylic acids and derivatives
	Creatinine	LMDB00180	Carboxylic acids and derivatives
	Dimethylamine	LMDB00037	Organonitrogen compounds
	Dimethyl sulfone	LMDB00459	Sulfonyl compounds
	Dimethylglycine	LMDB00039	Carboxylic acids and derivatives
	d-Mannose	LMDB00076	Organooxygen compounds
	Ethanol	LMDB00044	Organooxygen compounds
	Formate	LMDB00060	Carboxylic acids and derivatives
	Glucose	LMDB00048	Organooxygen compounds
	Glycerol	LMDB00055	Organooxygen compounds
	Glycine	LMDB00049	Carboxylic acids and derivatives
	Hippuric acid	LMDB00227	Benzene and substituted benzene derivatives
	Hypoxanthine	LMDB00067	Imidazopyrimidines
	Isobutyric acid	LMDB00357	Carboxylic acids and derivatives
	Isoleucine	LMDB00077	Carboxylic acids and derivatives
	l-Acetylcarnitine	LMDB00091	Fatty Acyl derivatives
	l-Alanine	LMDB00070	Carboxylic acids and derivatives
	l-Carnitine	LMDB00027	Organonitrogen compounds
	l-Glutamic acid	LMDB00063	Carboxylic acids and derivatives
	l-Glutamine	LMDB00202	Carboxylic acids and derivatives
	l-Histidine	LMDB00080	Carboxylic acids and derivatives
	l-Lactic acid	LMDB00084	Hydroxy acids and derivatives
	l-Leucine	LMDB00215	Carboxylic acids and derivatives
	l-Ornithine	LMDB00099	Carboxylic acids and derivatives
	l-Phenylalanine	LMDB00069	Carboxylic acids and derivatives
	l-Proline	LMDB00071	Carboxylic acids and derivatives
	l-Serine	LMDB00083	Carboxylic acids and derivatives
	l-Threonine	LMDB00074	Carboxylic acids and derivatives
	l-Lysine	LMDB00081	Carboxylic acids and derivatives
	Malonic acid	LMDB00217	Carboxylic acids and derivatives
	Methanol	LMDB00358	Organooxygen compounds
	Methionine	LMDB00221	Carboxylic acids and derivatives
	Oxoglutaric acid	LMDB00094	Keto acids and derivatives
	Pyruvic acid	LMDB00112	Keto acids and derivatives
	Sarcosine	LMDB00124	Carboxylic acids and derivatives
	Tyrosine	LMDB00068	Carboxylic acids and derivatives
	Urea	LMDB00131	Organic carbonic acids and derivatives
	Valine	LMDB00271	Carboxylic acids and derivatives
LC–MS/MS	SM (OH) C14:1	LMDB00624	Sphingolipids
	SM C16:0	LMDB00524	Sphingolipids
	SM C16:1	LMDB00656	Sphingolipids
	SM (OH) C16:1	LMDB00780	Sphingolipids
	SM C18:0	LMDB00569	Sphingolipids
	SM C18:1	LMDB01208	Sphingolipids
	SM C20:2	LMDB00626	Sphingolipids
	SM (OH) C22:1	LMDB00627	Sphingolipids
	SM (OH) C22:2	LMDB00628	Sphingolipids
	SM (OH) C24:1	LMDB00630	Sphingolipids
	Acetylornithine	LMDB00430	Carboxylic acids and derivatives
	Alpha-aminoadipic acid	LMDB00168	Carboxylic acids and derivatives
	Asymmetric dimethylarginine (ADMA)	LMDB00344	Carboxylic acids and derivatives
	C0 (Carnitine)	LMDB00027	Organonitrogen compounds
	C14:1 (tetradecenoylcarnitine)	LMDB01011	Fatty Acyl derivatives
	C2 (Acetylcarnitine)	LMDB00091	Fatty Acyl derivatives
	C3 (Propionylcarnitine)	LMDB00253	Fatty Acyl derivatives
	C4 (butyrylcarnitine)	LMDB00374	Fatty Acyl derivatives
	C5 (Valerylcarnitine)	LMDB00581	Fatty Acyl derivatives
	Carnosine	LMDB00010	Peptides
	Citrulline	LMDB00274	Carboxylic acids and derivatives
	Kynurenine	LMDB00214	Organooxygen compounds
	l-Aspartic acid	LMDB00085	Carboxylic acids and derivatives
	lysoPC a C14:0	LMDB00525	Glycerophospholipids
	lysoPC a C16:0	LMDB00526	Glycerophospholipids
	lysoPC a C16:1	LMDB00527	Glycerophospholipids
	lysoPC a C17:0	LMDB00571	Glycerophospholipids
	lysoPC a C18:0	LMDB00528	Glycerophospholipids
	lysoPC a C18:1	LMDB00409	Glycerophospholipids
	lysoPC a C18:2	LMDB00530	Glycerophospholipids
	lysoPC a C20:3	LMDB00533	Glycerophospholipids
	lysoPC a C20:4	LMDB00534	Glycerophospholipids
	lysoPC a C26:0	LMDB00653	Glycerophospholipids
	lysoPC a C26:1	LMDB01226	Glycerophospholipids
	Methionine sulfoxide	LMDB00373	Carboxylic acids and derivatives
	PC aa C32:2	LMDB01211^a^	Glycerophospholipids
	PC aa C36:0	LMDB01212^a^	Glycerophospholipids
	PC ae C36:0	LMDB01210^a^	Glycerophospholipids
	PC aa C36:6	LMDB01110^a^	Glycerophospholipids
	PC aa C38:0	LMDB01111^a^	Glycerophospholipids
	PC aa C38:6	LMDB01122^a^	Glycerophospholipids
	PC aa C40:1	LMDB01119^a^	Glycerophospholipids
	PC aa C40:2	LMDB01125^a^	Glycerophospholipids
	PC aa C40:6	LMDB01140^a^	Glycerophospholipids
	PC ae C40:6	LMDB00599	Glycerophospholipids
	Putrescine	LMDB00329	Organonitrogen compounds
	Serotonin	LMDB00120	Indoles and derivatives
	Spermidine	LMDB00311	Organonitrogen compounds
	Spermine	LMDB00310	Organonitrogen compounds
	Taurine	LMDB00115	Organic sulfonic acids and derivatives
	Total dimethylarginine	N/A	Carboxylic acids and derivatives
	Trans-Hydroxyproline (t4-OH-Pro)	LMDB00230	Carboxylic acids and derivatives
	Trimethylamine *N*-oxide	LMDB00278	Organonitrogen compounds
	Tryptophan	LMDB00279	Indoles and derivatives

Metabolites include those identified and quantified by NMR and LC–MS/MS from serum of healthy sheep assessed for pregnancy and litter size. Metabolite IDs identified by ^a^Refer to an isomer of that lipid. Note that total dimethylarginine does not have a LMDB ID since it consists of the sum of two metabolites (symmetrical and asymmetric dimethylarginine).

### Identifying PLS biomarkers via pairwise metabolomic comparisons

For the discovery phase of the study, the flocks were divided into six different groups based on their pregnancy and litter status (CNT = controls or open non-pregnant, PRG = pregnant, MLP = multiplet, SNG = singlets, TWN = twins, TRP = triplets). Each of the six groups were compared (pairwise) at each of the five different timepoints (7 days pre-breeding [− 7 day], day 0, 35, 50 and 70 post-breeding). In total 15 different pairwise comparisons were done over five timepoints (75 total comparisons). The outcomes from univariate and multivariate analyses of those comparison groups that yielded significant candidate biomarkers are presented in Tables 2, 3 and 4, respectively.

**Table 2:** Student’s t-test of four comparison groups from the discovery dataset.

T-test					
	Day − 7	Day 0	Day 35	Day 50	Day 70
CNT vs PRG	NS	NS	NS	Acetic acid, urea, SM (OH) C24:1, lysoPC a C26:0, lysoPC a C26:1, tryptophan, C3 (propionylcarnitine), carnosine, alpha-aminoadipic acid, putrescine, trimethylamine *N*-oxide, lysoPC a C18:2, hippuric acid, lysoPC a C14:0, l-arginine, lysoPC a C16:1	Urea, glycine, l-arginine, dimethylamine, formate, dimethyl sulfone, choline, acetic acid, 3-hydroxybutyric acid, acetoacetate, l-alanine, sarcosine, isobutyric acid, l-lysine, creatinine, pyruvic acid, d-mannose, l-serine
CNT vs MLP	NS	Kynurenine, l-ornithine	NS	Urea, acetic acid, SM (OH) C24:1, lysoPC a C26:0, l-arginine, C3 (propionylcarnitine), l-carnitine, tryptophan, lysoPC a C26:1, carnosine, putrescine	Urea, l-arginine, choline, glycine, acetic acid, dimethylamine, formate, 3-hydroxybutyric acid, dimethyl sulfone, acetoacetate, isobutyric acid, l-alanine, sarcosine, pyruvic acid, l-lysine, isoleucine
SNG vs TRP	NS	NS	l-Acetylcarnitine	Methionine	NS
TWN vs TRP	NS	NS	NS	Valine, l-lactic acid, Isobutyric acid	NS

Statistical analysis using t-test revealed significant (p-value < 0.05) serum metabolites of each comparison at five timepoints during the discovery phase. *NS* Not significant, *CNT* control open ewes, *PRG* pregnant ewes, *SNG* pregnant ewes that delivered one lamb, *TWN* pregnant ewes that delivered two lambs, *TRP* pregnant ewes that delivered more than two lambs. Day − 7 refers to 7 days prior to initiation of gestation and day 0 is the start of pregnancy.

**Table 3:** Volcano plot univariate analysis of four comparison groups from the discovery dataset.

Volcano plot					
	Day − 7	Day 0	Day 35	Day 50	Day 70
CNT vs PRG	Citric acid	SM C20:2, trans-hydroxyProline, kynurenine, total dimethylarginine	Acetone, total dimethylarginine, sarcosine, isobutyric acid, taurine, C3 (propionylcarnitine), methanol, putrescine	Acetic acid, SM (OH) C24:1, lysoPC a C26:0, lysoPC a C26:1, tryptophan, C3 (propionylcarnitine), putrescine, trimethylamine *N*-oxide, l-arginine, lysoPC a C16:1	Urea, glycine, l-arginine, dimethylamine, formate, dimethyl sulfone, choline, acetic acid, 3-hydroxybutyric acid, acetoacetate, sarcosine, l-lysine, acetone, dimethylglycine
CNT vs MLP	Citric acid^	l-Ornithine, kynurenine, trans-hydroxyProline, SM C20:2, total dimethylarginine	Acetone, l-ornithine, total dimethylarginine, isobutyric acid, taurine, trans-hydroxyProline, methanol, aspartic acid, C3 (propionylcarnitine), acetic acid, sarcosine, 3-hydroxyisovaleric acid	Acetic acid, SM (OH) C24:1, lysoPC a C26:0, l-arginine, C3 (propionylcarnitine), tryptophan, lysoPC a C26:1, carnosine, putrescine, lysoPC a C18:2, lysoPC a C16:1, lysoPC a C14:0, methionine-sulfoxide, spermidine, trimethylamine *N*-oxide	Urea, l-arginine, choline, glycine, acetic acid, dimethylamine, formate, 3-hydroxybutyric acid, dimethyl sulfone, acetoacetate, sarcosine
SNG vs TRP	Isobutyric acid	NS	l-Acetylcarnitine	Acetyl-ornithine, kynurenine, methionine	Choline, l-ornithine, ethanol
TWN vs TRP	Ethanol	C3 (propionylcarnitine), serotonin	Trans-hyrdoxyproline, kynurenine, hypoxanthine, acetone, formate, SM C20:2, lysoPC a C26:1	SM C20:2, valine, l-lactic acid, isobutyric acid	l-Ornithine, 3-methyl-2-oxovaleric acid, ethanol

Statistical analysis using volcano plot revealed significant (p-value < 0.05) serum metabolites of each comparison at five timepoints during the discovery phase. Metabolite noted with ^ has a tendency (p-value < 0.10). *NS* Not significant, *CNT* control open ewes, *PRG* pregnant ewes, *SNG* pregnant ewes that delivered one lamb, *TWN* pregnant ewes that delivered two lambs, *TRP* pregnant ewes that delivered more than two lambs. Day − 7 refers to 7 days prior to initiation of gestation and day 0 is the start of pregnancy.

**Table 4:** Partial least squares discriminant analysis (PLS-DA) analysis of four comparison groups from the discovery dataset.

PLS-DA VIP					
	Day − 7	Day 0	Day 35	Day 50	Day 70
CNT vs PRG	NS	NS	Putrescine, butyrate, sarcosine, l-ornithine, acetone, total dimethylarginine, ethanol, l-lysine, C3 (propionylcarnitine), taurine, methanol, trimethylamine *N*-oxide, isobutyric acid, aspartic acid, 3-hydroxyisovaleric acid	Acetic acid, urea, SM (OH) C24:1, lysoPC a C26:0, lysoPC a C26:1, tryptophan, C3 (propionylcarnitine), carnosine, alpha-aminoadipic acid, putrescine, trimethylamine *N*-oxide, lysoPC a C18:2, hippuric acid, lysoPC a C14:0, L-arginine	Urea, glycine, acetic acid, l-arginine, dimethyl sulfone, 3-hydroxybutyric acid, ethanol, l-lactic acid, l-lysine, sarcosine, dimethylamine, d-glucose, tyrosine, l-alanine, betaine
CNT vs MLP	Tendency	Urea, l-ornithine, l-lysine, acetoacetate, acetic acid, glycine, kynurenine, 3-hydroxybutyric acid, trans-hydroxyProline, total dimethylarginine, SM C16:0, taurine, l-threonine, methanol, butyrate	Acetic acid, l-ornithine, l-lysine, methanol, taurine, trimethylamine *N*-oxide, acetone, citric acid, sarcosine, ethanol, isobutyric acid, C0 (Carnitine), aspartic acid, butyrate, total dimethylarginine	Acetic acid, urea, l-arginine, tryptophan, carnosine, 3-hydroxybutyric acid, dimethyl sulfone, trimethylamine *N*-oxide, l-lysine, l-carnitine, lysoPC a C18:2, l-ornithine, hippuric acid, C0 (Carnitine), methanol	Urea, dimethylamine, l-arginine, glycine, dimethyl sulfone, choline, acetic acid, formate, 3-hydroxybutyric acid, l-alanine, isobutyric acid, acetoacetate, isoleucine, l-lysine, pyruvic acid
SNG vs TRP	NS	NS	NS	NS	Tendency
TWN vs TRP	NS	NS	NS	NS	NS

Multivariate statistical analysis of the discovery dataset using PLS-DA revealed top 15 metabolites that significantly (p-value < 0.05) differentiate between the two comparison groups at each timepoint. *NS* Not significant, *CNT* control open ewes, *PRG* pregnant ewes, *SNG* pregnant ewes that delivered one lamb, *TWN* pregnant ewes that delivered two lambs, *TRP* pregnant ewes that delivered more than two lambs. Day − 7 refers to seven days prior to initiation of gestation and day 0 is the start of pregnancy.

The data show that as ewes progress through gestation, the serum metabolome of pregnant ewes compared to open ewes, as well as pregnant ewes with different litter sizes, significantly diverges. Moreover, within each group, the blood metabolome significantly (p-value < 0.05) differed between each timepoint as determined by two-way ANOVA. Over the five timepoints tested, day 50 and day 70 yielded the most promising results. In particular, the volcano plot and the partial least squares discriminant analysis (PLS-DA) plot identified statistically significant metabolites that differentiated each group within each comparison. T-test results were most significant for the last two timepoints (days 50 and 70) between the most divergent comparison groups (CNT vs PRG and CNT vs MLP). Based on these results we then focused on identifying serum candidate biomarkers at day 50 and day 70 of gestation.

### Longitudinal assessment of significant metabolites during pregnancy

Longitudinal assessment of the t-test results (Table 2) revealed three significant metabolites (acetic acid, urea, and l-arginine) differentiating pregnant and open ewes at day 50 and day 70 of gestation. All the metabolites that were significantly different by day 50 (using a p-value threshold of < 0.05) for the CNT vs MLP groups were also significant in the CNT vs PRG comparison, except l-carnitine. Similarly, differentiating metabolites from day 70 (according to the t-test) of the CNT vs MLP groups were all similar to the CNT vs PRG group, except isoleucine. The similarities between these two comparisons were expected since the PRG group is composed of both MLP and SNG ewes.

Longitudinal assessment of the volcano plots (Table 3) among all pairwise comparison groups revealed that acetic acid was significantly different between the CNT vs MLP groups from day 35 of gestation. However, acetic acid was only significantly different from day 50 for the CNT vs PRG groups. At day 70 post-breeding, choline was significantly different in all comparison groups except the TWN vs TRP groups. We also observed that comparison of CNT against PRG and MLP at later timepoints of gestation shared the largest number of metabolite similarities among other data sets and comparisons.

Longitudinal assessment using PLS-DA and variable importance of projection (VIP; Table 4) showed that l-lysine and acetic acid were two of the 15 most differentiating metabolites throughout all timepoints of gestation (days 0, 35, 50 and 70) in the CNT vs MLP comparison. Three other metabolites (urea, 3-hydroxybutyric acid, and methanol) were also commonly observed in three of the four post-breeding timepoints (days 35, 50 and 70). Moreover, acetic acid and urea were the two highest scoring VIP metabolites on day 50 and day 70 in both the CNT vs PRG and CNT vs MLP comparisons. This further confirms the trend observed in univariate analyses and underlines how the CNT group, when compared against the PRG and MLP groups, typically shared more metabolic similarities in later pregnancy timepoints.

Temporal trends were then investigated. For the CNT vs PRG comparison, one group of significantly altered metabolites at day 50 was identified (acetic acid, l-arginine, SM (OH) C24:1, lysoPC a C26:0, lysoPC a C26:1, tryptophan, C3 [propionylcarnitine], putrescine, trimethylamine *N*-oxide,), while another group was identified at day 70 (acetic acid, l-arginine, urea, glycine, dimethylamine, dimethyl sulfone, 3-hydroxybutyric acid, sarcosine, l-lysine). These metabolites were consistently identified by all statistical analyses.

Temporal comparison of the CNT group against the MLP group at days 0 and 35 identified l-ornithine as a significantly altered metabolite. l-ornithine was found to be significant in all analyses for both timepoints. Acetic acid was another significantly altered metabolite at day 35. At day 50 of gestation, the metabolites that exhibited the greatest difference included acetic acid, l-arginine, tryptophan and carnosine. At day 70, nine other significantly altered metabolites were identified, including urea, l-arginine, choline, glycine, acetic acid, dimethylamine, formate, 3-hydroxybutyric acid, dimethyl sulfone and acetoacetate. In contrast, we did not identify any temporal pattern using univariate or multivariate statistical analyses of the SNG vs TRP groups or the TWN vs TRP groups.

### Candidate biomarkers of ewe pregnancy

To identify candidate biomarkers of ewe pregnancy, we compared the CNT ewes against all other pregnant ewes regardless of their litter size (PRG). To seek further confirmation and examine the extremes in terms of litter size, we removed the SNG ewes from the PRG dataset and also compared the CNT and MLP ewes. The advantage of the latter comparison is that the outcome biomarkers could help inform producers not only if the animal is pregnant but also that the ewe is expected to deliver more than one lamb. A detailed summary of the results is presented in Table 5. An additional table (Supplementary Table 1) shows the average individual concentration values (at day 50) for each of the metabolites and conditions mentioned in Tables 2, 3, 4 and 5. A complete list of metabolites identified in sheep serum and their corresponding concentrations at all timepoints reported in this study is also listed in the open access LMDB (the Livestock Metabolome Database; www.lmdb.ca). We identified no statistically useful serum biomarkers until day 35 of gestation when comparing the CNT group with the PRG group. However, at day 50 of the CNT vs PRG comparison, we identified a panel of five metabolites (methanol, l-carnitine, d-glucose, l-arginine, and urea; with an area under the receiver operating characteristic curve (AU-ROC) = 0.76) that could serve as candidate biomarkers for detecting pregnant ewes. At day 70, we identified a panel of two metabolites for ewe pregnancy that had an AU-ROC of close to 1.0 with very high statistical significance (p-value < 0.001). Comparing the CNT and MLP groups, we identified no useful biomarkers at day-7, while the other four timepoints revealed potentially useful biomarkers. The AU-ROC value and statistical significance of the biomarkers improved substantially later in the gestation, i.e., at day 70. Among the different timepoints assessed, day 50 had the largest panel of biomarkers, and these biomarkers were identical to the candidate biomarkers found at day 50 of the CNT vs PRG comparison. Given the value of detecting PLS at the earliest timepoint in gestation, a logistic regression equation was developed for the candidate biomarkers found at day 50 using the CNT vs PRG comparison. This equation is given below:

1 $$\begin{aligned} {\text{logit}}\left( {\text{P}} \right) \, & = {\text{ log}}\left( {{\text{P }}{/}\left( {{1} - {\text{P}}} \right)} \right) \, = { 1}.{599 } + { 1}.{217}\,{\textsc{l}}{\text{{-}arginine }} + { 2}.0{95}\,{\text{urea }} \\ & \quad + { 1}.{222}\,{\textsc{l}}{\text{{-}carnitine }} + \, 0.{137}\,{\text{methanol }}{-} \, 0.{5}0{5}\,{\textsc{d}}{\text{{-}glucose,}} \\ \end{aligned}$$

where P is the probability of pregnancy occurring with a cut-off of 0.81. Because the concentrations of the metabolites used in the CNT vs PRG comparison were sum normalized, log transformed and Pareto scaled, the metabolite values used in the equation must be adjusted. These adjustments are provided in Table 6. This same logistic regression equation was later used to predict the pregnancy status of ewes in the validation phase.

**Table 5:** Receiver operating characteristics (ROC) analysis of the comparison groups in the discovery and validation datasets.

ROC						
Discovery phase						Validation phase
	Day − 7	Day 0	Day 35	Day 50	Day 70	Day 50
CNT vs PRG	NA	NA	NA	Methanol, l-carnitine, d-glucose, l-arginine, urea	Urea, glycine	Methanol, l-carnitine, d-glucose, l-arginine, urea
	NA	NA	NA	AU-ROC = 0.76 p < 0.10	AU-ROC = 0.98 p < 0.001	AU-ROC = 0.90 p < 0.05
CNT vs MLP	NA	l-Ornithine, choline	Acetone, l-ornithine, C0, total dimethylarginine	Methanol, l-carnitine, d-glucose, l-arginine, urea	Choline, urea, l-arginine, glycine	Methanol, l-carnitine, d-glucose, l-arginine, urea
	NA	AU-ROC = 0.79 p < 0.05	AU-ROC = 0.73 p < 0.05	AU-ROC = 0.76 p < 0.05	AU-ROC = 0.97 p < 0.01	AU-ROC = 0.93 p < 0.001
SNG vs TRP	Choline, l-carnitine, l-phenylalanine	C4, l-threonine, trans-hydroxyproline	l-Acetylcarnitine, l-carnitine, trans-hydroxyproline	Methionine, l-carnitine	Choline, d-glucose, l-phenylalanine	Methionine, l-carnitine
	AU-ROC = 0.80 p < 0.05	AU-ROC = 0.74 p < 0.05	AU-ROC = 0.76 p < 0.10	AU-ROC = 0.78 p < 0.05	AU-ROC = 0.81 p < 0.05	AU-ROC = 0.84 p < 0.001
TWN vs TRP	Hypoxanthine, l-phenylalanine, choline, l-carnitine, creatinine	Serotonin, C3	Hypoxanthine, trans-hydroxyproline, kynurenine	Isobutyric acid, l-lactic acid, l-carnitine, valine, tyrosine, methanol	Hypoxanthine, l-phenylalanine, l-carnitine, isobutyric acid	Isobutyric acid, l-lactic acid, l-carnitine, valine, tyrosine, methanol
	AU-ROC = 0.77 p < 0.10	AU-ROC = 0.74 p < 0.05	AU-ROC = 0.75 p < 0.05	AU-ROC = 0.66 p < 0.10	AU-ROC = 0.77 p < 0.05	AU-ROC = 0.81 p < 0.05

Candidate biomarkers were evaluated during all five timepoints of the discovery phase and day 50 of gestation was the best timepoint to reveal candidate biomarkers of ewe PLS. Therefore, biomarker analysis was pursued for only day 50 of gestation in the validation phase. The panel of metabolites that reached an area-under-the-curve (AU-ROC) of at least 0.65 or were significant (p-value < 0.05) were considered as candidate biomarkers in the discovery phase and were confirmed as biomarkers if the AU-ROC and p-value improved in the validation analysis. *NS* Not significant, *NA* biomarker not available, *CNT* control open ewes, *PRG* pregnant ewes, *SNG* pregnant ewes that delivered one lamb, *TWN* pregnant ewes that delivered two lambs, *TRP* pregnant ewes that delivered more than two lambs. Day − 7 refers to seven days prior to initiation of gestation and day 0 is the start of pregnancy.

**Table 6:** Biomarker concentrations adjusted for calculation in the logistic regression.

	CNT vs PRG	SNG vs TRP	TWN vs TRP
Methanol	Log_2_([methanol]/4901.36) − 7.13)/1.08	N/A	Log_2_([methanol]/2261.69) + 0.25)/0.07
l-Carnitine	Log_2_([l-carnitine]/3733.21) − 6.76)/0.56	Log_2_([l-carnitine]/39.70) + 0.98)/0.10	Log_2_([l-carnitine]/1961.53) + 0.0.26)/0.03
d-Glucose	Log_2_([d-glucose]/384,197.32) − 6.76)/0.57	N/A	N/A
l-Arginine	Log_2_([l-arginine]/21,202.62) − 6.85)/0.81	N/A	N/A
Urea	Log_2_([urea]/205,076.40) − 6.80)/0.61	N/A	N/A
Methionine	N/A	Log_2_([methionine]/30.22) + 0.98)/0.12	N/A
Isobutyric Acid	N/A	N/A	Log_2_([isobutyric acid]/669.83) + 0.26)/0.03
l-lactic acid	N/A	N/A	Log_2_([l-lactic acid]/145,410.12) + 0.26)/0.04
Valine	N/A	N/A	Log_2_([valine]/10,719.58) + 0.26)/0.03
Tyrosine	N/A	N/A	Log_2_([tyrosine]/3242.95) + 0.26)/0.05

Raw concentration of each metabolite (indicated using square brackets) is converted based on the following formula and the resulting value is used in the corresponding logistic regression equation.

### Candidate biomarkers of ewe litter size

Comparisons were made of CNT vs MLP groups (to identify pregnant ewes that deliver more than one lamb), SNG vs TRP groups (pregnant ewes that deliver a single or more than two lambs) and TWN vs TRP groups (pregnant ewes that deliver a twin or more than two lambs). A detailed summary of results is presented in Table 5. Candidate biomarkers were identified at all five timepoints for the SNG vs TRP comparison. This comparison revealed three to four candidate biomarkers at each timepoint with AU-ROC values varying from a low of 0.74 on day 0 to a high of 0.81 on day 70. All biomarkers were statistically significant except for the markers identified for day 35, which only had a statistical tendency. l-carnitine was the most frequently observed candidate biomarker, appearing at days − 7, 35 and 50. Since day 50 of gestation was the earliest timepoint to detect pregnancy, this timepoint was used to develop a logistic regression equation for the panel of candidate biomarkers (methionine and l-carnitine) of the SNG vs TRP comparison. This equation is given below:

2 $${\text{logit}}\left( {\text{P}} \right) \, = {\text{ log}}\left( {{\text{P}}/\left( {{1} - {\text{P}}} \right)} \right) \, = \, 0.{211 }{-}{ 4}.{464}\,{\text{methionine }} + { 4}.{393}\,{\textsc{l}}{\text{{-}carnitine,}}$$

where P is the probability of delivering more than two lambs with a cut-off of 0.70. Because the concentrations of the metabolites used in this study were median normalized, cube root transformed and Pareto scaled, the metabolite values must be adjusted. These adjustments are provided in Table 6.

With regard to the TWN vs TRP group comparison, l-carnitine was also identified as the most frequently recurrent metabolite at all timepoints. For this comparison group, biomarkers at days − 7 and day 50 only had a statistical tendency, while other timepoints had statistically significant biomarkers. All AU-ROC values were below 0.80 and most panels consisted of a relatively larger number of metabolites. The candidate biomarkers (isobutyric acid, l-lactic acid, l-carnitine, valine, tyrosine, and methanol) identified for the TWN vs TRP comparison groups at day 50 of gestation were used to develop a logistic regression model as follows:

3 $$\begin{aligned} {\text{logit}}\left( {\text{P}} \right) \, & = {\text{ log}}\left( {{\text{P }}{/} \left( {{1} - {\text{P}}} \right)} \right) \, = \, - 0.{124 } + \, 0.{4}0{6}\,{\text{isobutyric}}\,{\text{acid }}{-} \, 0.{388}\,{\textsc{l}}{\text{{-}lactic}}\,{\text{acid }} \\ & \quad {-} \, 0.{771}\,{\textsc{l}}{\text{{-}carnitine }} + \, 0.{593}\,{\text{valine }} + \, 0.{144}\,{\text{tyrosine }} + \, 0.{683}\,{\text{methanol,}} \\ \end{aligned}$$

where P is the probability of triplets over twins occurring with a cut-off of 0.57. Because the concentrations of the metabolites used in this study were sum normalized, cube root transformed and auto scaled, the metabolite values used in the equation must be adjusted. These adjustments are provided in Table 6. The above two equations were later used to predict litter size status of pregnant ewes in the validation phase.

### Validation phase

Given that we determined the ideal time to assess PLS in ewes via serum metabolomics was at day 50 post-breeding, the sample collection for the validation phase was conducted only at day 50 of gestation. This section describes the validation of the same panel of day 50 candidate biomarkers, and the prediction of the validation dataset using the logistic regression equations developed in the discovery phase. In conducting this validation phase, we looked at approximately twice the number of samples analyzed in the discovery phase from commercial flocks located in different regions and under different management practices (in two of the top sheep producing provinces in Canada, Alberta and Ontario).

### Validated biomarkers of ewe pregnancy

**Figure 1:**
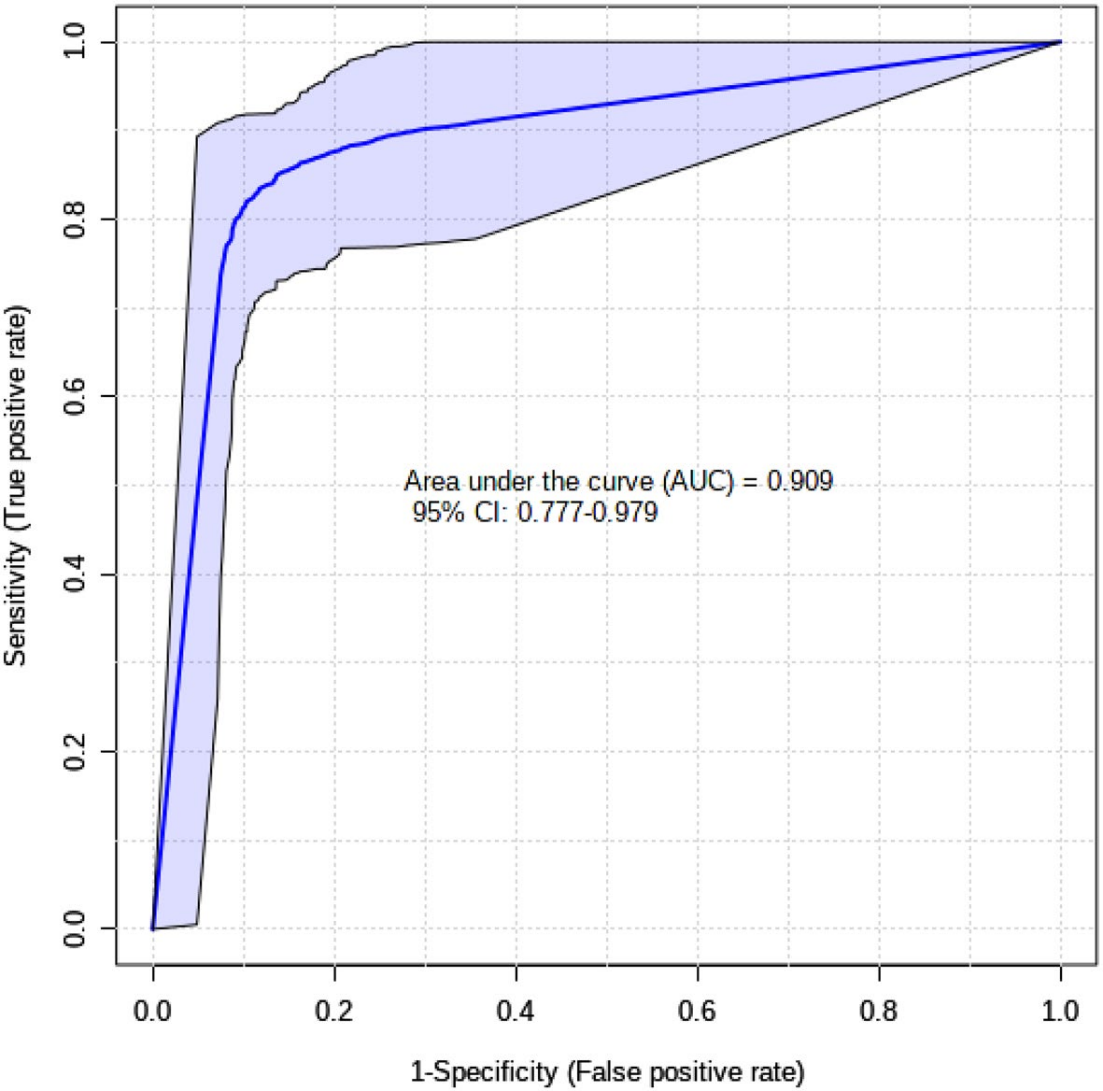
Receiver operating characteristics (ROC) curve of biomarkers of sheep pregnancy. The panel of five metabolites (methanol, l-carnitine, d-glucose, l-arginine, urea) from the CNT vs PRG comparison were selected as significant (p-value < 0.05) biomarkers of sheep pregnancy.

Statistical analyses of the validation dataset for the five candidate biomarkers of pregnancy (presented previously) improved the AU-ROC to ≥ 0.90 (Fig. 1) and the p-value to < 0.05 (Table 5). Methanol, l-carnitine, d-glucose, l-arginine, and urea were confirmed to be robust biomarkers to detect ewe pregnancy at day 50 of gestation. Supplementary Fig. 1 shows the boxplots for these five metabolites comparing their normalized/scaled values between pregnant and non-pregnant ewes. The same logistic regression model (Eq. 1) presented for the candidate biomarkers in the discovery phase was used to predict the pregnancy status of the validation dataset. This regression model was successful in making predictions with a sensitivity of 69% and a specificity of 85%.

### Validated biomarkers of ewe litter size

**Figure 2:**
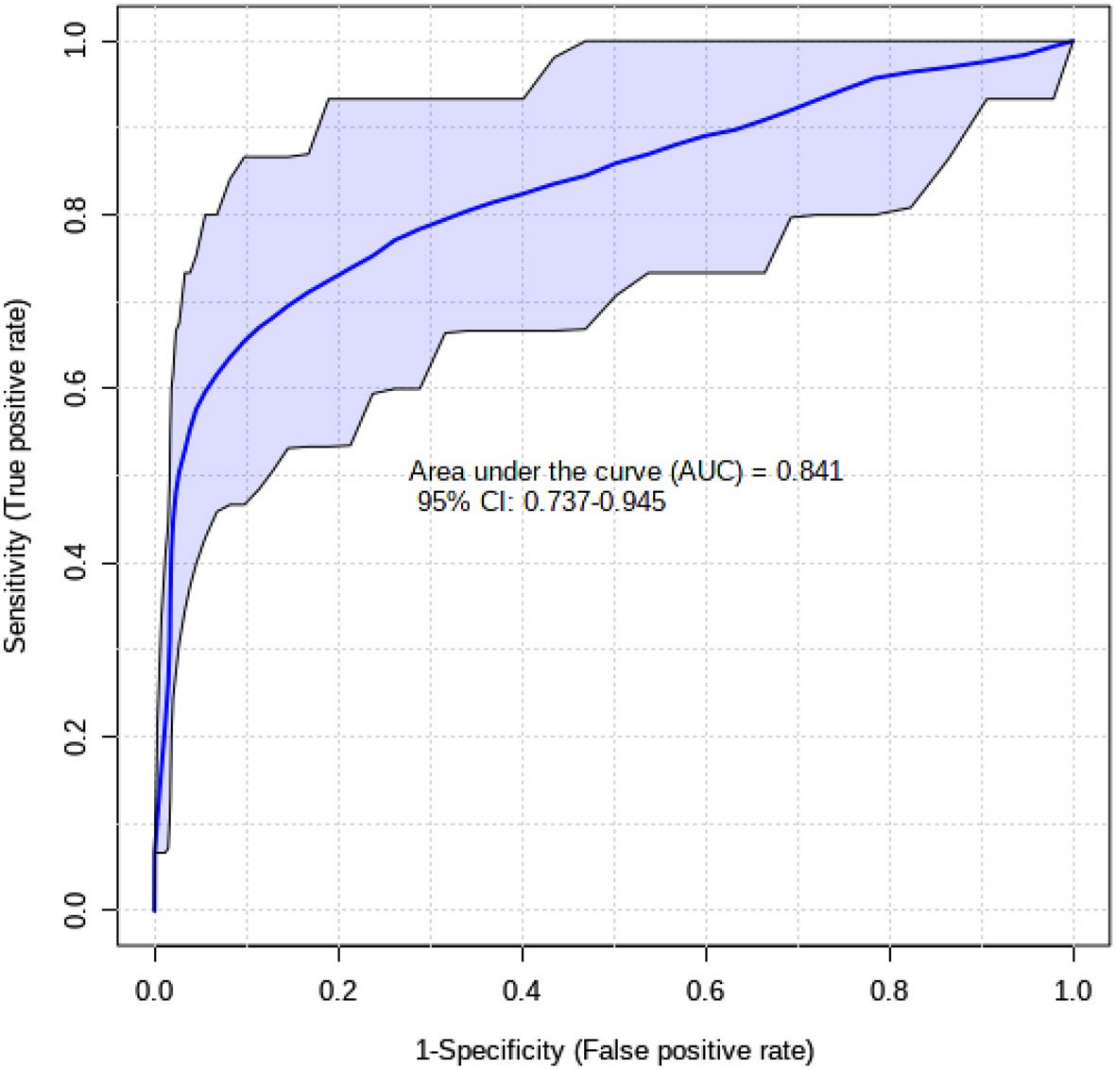
Receiver operating characteristics (ROC) curve of biomarkers of pregnant ewes with a single or more than two lambs. The comparison of SNG vs TRP groups identified methionine and l-carnitine as significant (p-value < 0.001) biomarkers that would identify ewes that carry a single lamb or those that carry more than two lambs.

The AU-ROC value for candidate biomarkers (methionine and l-carnitine) of SNG vs TRP improved from 0.78 in the discovery phase to 0.84 in the validation set (Fig. 2). This was accompanied by improved significance from a p-value < 0.05 to a p-value < 0.001 (Table 5). Therefore, methionine and l-carnitine appear to be robust biomarkers of ewe litter size. Supplementary Fig. 2 shows the box plots for these two metabolites comparing their normalized/scaled values between SNG and TRP pregnancies. The same logistic regression model (Eq. 2) developed in the discovery phase to distinguish SNG vs TRP was used in the validation dataset. The regression model was successful in predicting litter size (SNG vs. TRP) with a sensitivity of 56% and a specificity of 91%.

**Figure 3:**
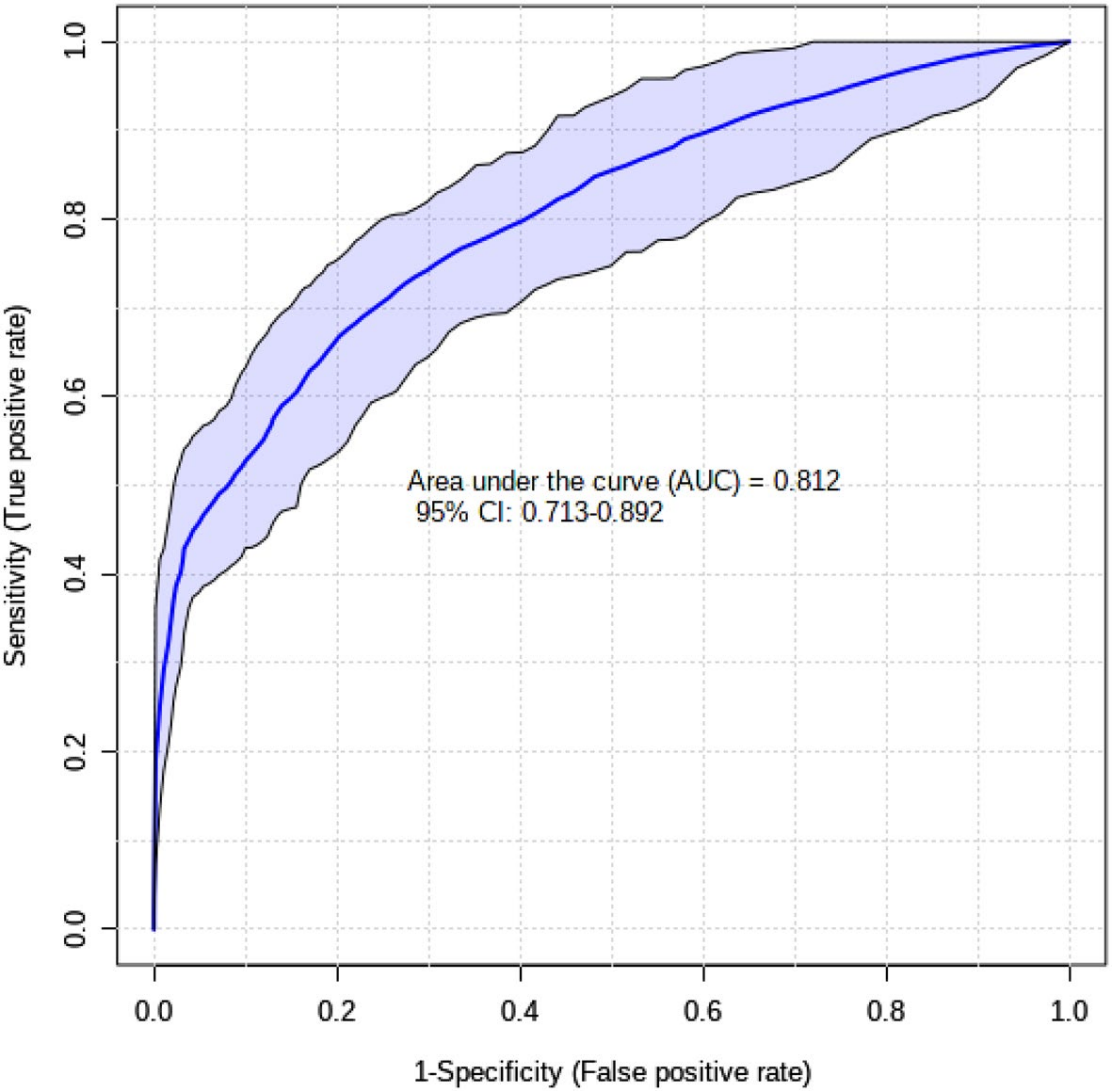
Receiver operating characteristics (ROC) curve of biomarkers of pregnant ewes with twin or triplet lambs. A panel of six metabolites (isobutyric acid, l-lactic acid, l-carnitine, valine, tyrosine, methanol) from comparing TWN vs TRP groups were identified as significance (p-value < 0.05) biomarkers of pregnant ewes that carry multiple lambs.

The candidate biomarkers (isobutyric acid, l-lactic acid, l-carnitine, valine, tyrosine, and methanol) identified for the TWN vs TRP comparison also reached statistical significance with an improved AU-ROC of 0.81 (Fig. 3). These compounds were confirmed as robust biomarkers of ewe litter size. The same logistic regression model (Eq. 3) was used for the panel of candidate biomarkers of TWN vs TRP comparison groups developed in the discovery phase to predict the validation dataset. Supplementary Fig. 3 shows the box plots for these six metabolites comparing their normalized/scaled values between TWN and TRP pregnancies. This regression model was successful in predicting litter size (TWN vs. TRP) with a sensitivity of 66% and specificity of 85%.

Biomarkers of pregnancy overlapped with those of the CNT versus MLP comparison groups indicating that if a ewe tests positive for the panel, not only is she pregnant but she is also expected to carry multiple fetuses. On the other hand, if the animal tests negative, she is not pregnant. To get a more precise measure of the litter size, further evaluation of the pregnant ewe’s blood using the other panels of litter size biomarkers will likely be required. Therefore, if a pregnant ewe tests positive for the triplet biomarker panel (methionine, l-carnitine), the ewe is expected to deliver more than two lambs while a negative test does not necessarily indicate that the ewe will deliver a single lamb. On the other hand, pregnant ewes that test negative for biomarkers of twin vs triplet biomarker panel (isobutyric acid, l-lactic acid, l-carnitine, valine, tyrosine, and methanol) are expected to deliver twins.

## Discussion

Over the past decade, livestock metabolomics research has gained considerable momentum. Currently the number of papers being published on the subject is almost doubling every 2 years. However, sheep metabolomics is still lagging behind the research activities for other livestock species such as cattle and pigs. For this reason, we focused on further characterizing the sheep metabolome and identifying candidate biomarkers associated with production traits of high economic value such as residual feed intake, carcass merit^23^ and reproductive performance. In this study, we examined sheep serum using NMR and LC–MS/MS-based metabolomics to identify robust and useful metabolite biomarkers of PLS. The initial step involved profiling the sheep serum metabolome during the first half of pregnancy. In doing so, we identified and quantified a total of 107 serum metabolites. Although no new sheep serum metabolites were identified (after comparison to the data in the LMDB^12^), the proportion of quantified sheep serum metabolites in the LMDB were increased from 49 to 52%. Data from this experimental work also adds to the reference values obtained from healthy pregnant sheep in the LMDB. Moreover, the study provides quantitative information about the metabolic dynamics of the ewe serum metabolome from seven days prior to breeding to day 70 of gestation. These data are now publicly accessible in the LMDB (www.lmdb.ca).

The central objective of this study was to identify serum metabolite biomarkers for sheep PLS using high-throughput, quantitative metabolomic platforms. As far as we are aware, this is the first study to identify non-hormonal metabolite biomarkers of both pregnancy and litter size, and to provide logistic regression models to predict pregnancy status in domestic sheep. It is important to note, however, that there are other compounds or biomarkers that have shown promise for assessing ewe PLS. These include genes, proteins and metabolites, some of which are described below.

### Previously identified PLS biomarkers

Efforts to identify specific gene transcript levels and genetic markers for sheep PLS have been previously described. For example, changes in the expression levels of the interferon-tau-stimulated gene in the thymus^24^ and endometrium^25^ have been found to signal pregnancy at early gestation. There are also a number of studies on genes responsible for sheep litter size^26^. The Booroola gene, located on ovine chromosome 6, has a major impact on ovulation rate and is a major determining factor for litter size in sheep^27^. This gene has at least 23 different variants. Certain Booroola variants increase follicle sensitivity to the follicle-stimulating hormone, thereby inducing a faster follicle maturation^28^. Moradband et al.^29^ found that heterozygotes in the Iranian Baluchi sheep breed had increased the litter size. Ewes that are homozygous for the variant almost double their ovulation rate. However, their lambs have a low survival rate with a lower growth rate and weaning rate^28^.

The Booroola gene is associated with the bone morphogenetic protein receptor 1B (BMPR-1B^26^). Increased blood concentrations of the BMPR-1B protein have been reported to benefit follicular development, yielding better ovulation and increased litter size^30^. A separate study that evaluated proteins in the follicular fluid (FF) of ewes found that the FF of larger follicles compared to smaller follicles had increased glucose and cholesterol concentrations, but lower concentration of triglycerides, lactate, alkaline phosphatase and lactate dehydrogenase^31^. These metabolites and proteins appear to be correlated with ovulation rate, suggesting their relevance to prolific ewes and the litter they carry. In another study, Koch et al.^32^ used MS-based proteomics to identify 15 signature proteins from the uterine luminal fluid of ewes as indicators of pregnancy and involved with embryonic growth, immune regulation and nutritional needs. As yet, none of these protein markers have been rigorously validated by ROC curve analysis and none are commercially used in sheep PLS testing.

Another example of a protein biomarker in pregnant ewes is the pregnancy-associated glycoprotein (PAG). The PAG is a placental-secreted factor that is detected in maternal serum upon implantation of the fetus onto the endometrium. This protein can be measured as early as 30 days in gestation^33^, with increasing concentrations as the pregnancy progresses^34^. Pregnancy specific protein B (PSPB is a form of PAG that is released by the fetus to maintain the corpus luteum^35^. Also, PSPB along with other PAGs increases with increasing number of fetuses carried by the ewe (Pickworth et al. ^36^). However, PSPB is breed-specific (Redden and Passavant^37^) which limits its application for all sheep breeds. Generally, PAGs are also positively correlated with maternal serum P4 levels^34^. In a study by Karen et al.^13^, blood PAG had 93.5% sensitivity for detecting pregnancy at day 22 of gestation, however, their results were skewed by the abnormally low (17%) pregnancy rate of the flock.

In addition to genetic and protein biomarkers of sheep PLS, a number of metabolite biomarkers have also been explored. Progesterone is a promising example of a hormonal metabolite biomarker that could be used for assessing sheep PLS. Progesterone is predominantly produced by the CL at the beginning of gestation and later (day 50 onwards) is produced by the placenta to maintain the pregnancy^34,38^. The concentration of P4 in ewe blood increases over the course of gestation and has been used as an indicator of pregnancy, as well as placental and fetal wellbeing^34^. However, identifying ewe PLS through measurements of P4 concentrations at around days 50–80 of gestation has a sensitivity varying between 65 and 85% and a specificity between 65 and 93%^21,39^. While potentially promising, blood P4 concentrations are not considered sufficiently accurate indicators of non-pregnant ewes^13^ and are not useful for differentiating ewes based on litter size^21^. Furthermore, LC–MS-based metabolomic analysis of a panel of eight steroid hormones, including P4, in our own sheep serum samples (n = 94), showed no improvement in biomarker performance when using P4 independently or in combination with non-hormonal metabolites to detect sheep PLS status (unpublished data). Another steroid hormone, estradiol, has also been used for detecting litter size after 50 days into gestation^40^. Despite P4 and estradiol being significant reproductive hormones and associated with ewe PLS, to date there is insufficient evidence and validation based on ROC analysis or regression modeling to make these hormones useful for assessing sheep PLS status^41^.

Other (non-hormonal) metabolites have also been identified as potential pregnancy markers in other livestock species. A recent study of pregnant buffaloes identified five milk metabolites detected by LC–MS on day 18 after artificial insemination as candidate biomarkers of pregnancy^15^. Likewise, in beef cattle, four plasma metabolites were detected by NMR at day 40 of gestation^16^. These reports suggest that measurement of non-hormonal metabolites may serve as an indirect means of pregnancy and/or litter size detection in ruminants.

To date, few studies have reported non-hormonal metabolites associated with sheep PLS. Sun et al.^17^ used NMR to investigate pregnant ewe metabolism in relation to in utero fetal growth at four timepoints from day 50 of gestation onwards. They reported 13 serum metabolites that are associated with protein and lipid metabolism of twin-bearing pregnant ewes. In another study using MS-based analysis of FF and ovarian vein serum in the Han sheep breed^42^, a total of eight metabolites (glucose 6-phosphate, glucose 1-phosphate, aspartate, asparagine, glutathione oxidized, cysteine-glutathione disulfide, γ-glutamylglutamine, and 2-hydroxyisobutyrate) were significantly associated with ewe litter size. Another recent metabolomic study using LC–MS/MS revealed that sphingolipid and amino acid metabolism is important for maintaining the uterine environment to increase embryo survival rate^43^. In addition to these studies, there are a few other reports that measured individual metabolites in pregnant sheep^18–20,22^. None of these studies identified or rigorously assessed the reported metabolites as robust PLS biomarkers. Overall, existing data suggests that individual genes, proteins and metabolites may be useful for assessing sheep PLS. However, as of yet, there have been no metabolomic studies that have attempted to rigorously identify and validate a panel of readily accessible non-hormonal metabolite blood biomarkers for assessing sheep PLS.

A common feature of the serum biomarkers presented in this study is that all are detectable by NMR spectroscopy. While the identification and validation of a set of useful sheep PLS biomarker panels was our primary interest in this study (see Table 5), we also believe it is important to provide some biological context and to suggest how some of these metabolites may play a role in sheep pregnancy. Indeed, the biological role of some of these metabolites appears to tie in with the reproductive physiology of sheep. However, some metabolites have not previously been identified as having a role in pregnancy, litter size or gestation and so it is difficult to understand their biological context. The following section further discusses the known biological relevance of each metabolite biomarker identified in this study. It also elaborates on the potential impact that these biomarkers may have for the sheep industry.

### Potential biological roles of the PLS biomarkers identified in this study

L-arginine is an essential amino acid that is known to be important for successful pregnancy. At day 50 of gestation, l-arginine was significantly (p-value < 0.05; Table 2) elevated in pregnant ewes (214 ± 85 µM) relative to non-pregnant controls (174 ± 78 µM). Arginine appears to play a role in a number of physiological pathways related to pregnancy. Luther et al.^44^ provided pregnant ewes with l-arginine supplementation and observed enhanced ovarian function along with elevated numbers of viable fetuses. The same study identified a direct positive correlation between l-arginine and P4, leading to improved pregnancy maintenance and early embryonic growth. Our results appear consistent with these reports and show that pregnant ewes as well as ewes that delivered more lambs had a higher serum concentration of l-arginine. Furthermore, maternal administration of this amino acid in the later portion of gestation has been shown to increase lamb birth weight, enhance blood flow and increase nutrient transport to the fetus through synthesis of nitric oxide^45,46^. l-arginine also improves pancreatic and brown adipose tissue growth during fetal development^47^, and increases post-partum brown fat storage and the survivability of female lambs^48^. Serum l-arginine is associated with improved post-partum weaning weight and the weaning rate of lambs^49^. Administering this amino acid to prolific ewes further improves the lambing rate by nearly 60%, increases lamb birth weight by over 20% without negatively impacting maternal body weight, and decreases lamb mortality rate at birth by more than 20%^50^.

Another metabolite identified as a strong biomarker of litter size was urea. At day 50 of gestation, the average urea concentration was significantly (p-value < 0.001) lower in pregnant ewes (1823 ± 667 µM) compared to open ewes (2518 ± 871 µM). Urea is a source of nitrogen for rumen microbes and is produced through the degradation of amino acids. Elevated blood concentration of urea in ewes seems to reduce conception and pregnancy rate^51^. Likewise, high concentrations of circulating urea have adverse impacts on embryonic development^52^. Our results are in agreement with these findings as pregnant ewes as well as ewes with a greater litter size have a lower concentration of blood urea compared to non-pregnant ewes.

One of the more interesting biomarkers we identified for litter size was methionine. We found that the average methionine serum concentration was significantly lower (28 ± 9 µM, p-value < 0.001) in pregnant ewes that delivered more than two lambs compared to ewes that delivered just one lamb (33 ± 9 µM). Methionine is an essential amino acid that plays an important role in general animal performance^53^, as well as the growth and development of lambs in early life^54^. Methionine is also a methyl group supplier for epigenetic alteration of DNA, especially in late gestation^55^. Indeed, Sinclair and associates^56^ reported widespread epigenetic alterations in progeny, mostly male lambs, resulting from restricted supply of dietary methionine to the pregnant dam. Alterations to the genome induced by metabolites such as methionine are responsible for modification of health-related phenotypes, cell growth, host immunity, and protein production^56–59^.

l-lactic acid is another biomarker of litter size that is traditionally associated with muscle metabolism. However, during pregnancy its concentration increases with the progression of gestation^60^. Average l-lactic acid concentration was significantly higher (3293 ± 1948 µM, p-value = 0.01) in pregnant ewes that delivered more than two lambs compared to ewes that delivered only two lambs (2432 ± 989 µM). Lactate can be used as an alternative source of energy by the fetal brain^61^. Therefore, a ewe with a higher number of fetuses is expected to have a higher concentration of serum l-lactic acid.

Valine is another biomarker we found to be associated with ewe litter size, and it decreased with increasing number of lambs. The average valine serum concentration was significantly higher (219 ± 74 µM, p-value = 0.007) in TWN versus TRP (191 ± 64 µM) pregnant ewes. This metabolite is a branched-chain amino acid that stimulates protein synthesis in fetal muscle^62,63^. Therefore, ewes that deliver three or more lambs and have an overall higher fetal protein synthesis compared to those that deliver twins are expected to have a higher utilization of this amino acid and lower concentration in the serum. Branched-chain amino acids are also integral to the immune system by supporting the growth of lymphocytes and natural killer cells to remove viral infections^64^. Pregnant ewes are more prone to immune challenges and an increased number of fetuses increases immune vulnerability of the ewe^65,66^. Therefore, ewes that have the largest litter size, i.e., triplets vs twins, are expected to draw more valine from the maternal serum, which aligns with our results.

### Comparison to ultrasonography

The current gold standard for sheep PLS assessment is ultrasonography. Ultrasound is mostly used to determine pregnancy status (open vs pregnant). However, certain experienced ultrasound operators can detect the number of fetuses in pregnant ewes as early as approximately 40–45 days of pregnancy and onwards (based on industry data in Canada). In fact, our field observations indicate that most Canadian ultrasound technicians identify litter size as one fetus or more than one. Ultrasound scanning is relatively rapid (2–5 min/ewe) and costs CAD$5–8/ewe (depending on the location of the farm, travel required for the operator to reach the farm, and the number of ewes being scanned). All sheep used in this study were characterized via ultrasound analysis by trained technicians at day 50 of pregnancy.

Using records from 166 ewes with complete data from ultrasound scanning and corresponding pregnancy outcome, we determined that the sensitivity of ultrasound was 0.55, the specificity was 0.70 and the AU-ROC of using ultrasonography for pregnancy detection was 0.65. With regard to ultrasonography results for litter size, we found that for distinguishing SNG vs TRP, the sensitivity was 0.51 while the specificity was 0.18. With regard to distinguishing TWN vs TRP, the sensitivity of ultrasonography was 0.43 while the specificity was 0.18. It is noteworthy that the consistency of ultrasound prediction varied between farms mainly due to the expertise and experience of the technician who tended to underestimate singles and triplets while overestimating twins. Comparing our metabolomics results to these ultrasound measurements (Table 7) serum metabolite markers performed better than ultrasonography by 24% in terms of AU-ROC, 20% in terms of sensitivity, and 18% in terms of specificity for detecting ewe pregnancy. Likewise, if we compare our predictive biomarker panels for detecting litter size against ultrasonography, metabolite panels performed 9–35% better in terms of sensitivity and nearly 80% better in terms of specificity for predicting litter size. These results indicate serum metabolite measurements are significantly more accurate than ultrasound in detecting and assessing sheep PLS in this study.

**Table 7:** Performance comparison of metabolomic biomarkers and ultrasonography.

	Ultrasonography CNT vs PRG	Ultrasonography SNG vs MLP	CNT vs PRG	SNG vs TRP	TWN vs TRP
Sensitivity	0.56	0.87	0.69	0.56	0.66
Specificity	0.70	0.53	0.85	0.91	0.85
AU-ROC	0.65	0.68	0.85	0.82	0.80

Sensitivity and specificity and the ability to predict sheep PLS is compared between ultrasonography and regression models of blood metabolite biomarkers. Most biomarker panels offer a higher sensitivity and specificity than that of ultrasound diagnosis of PLS. The values calculated for ultrasound are for detecting pregnancy status (CNT vs PRG) and whether the pregnant ewes carry a single fetus or more (SNG vs MLP) while, the biomarker panels also identify the specific number of the litter (i.e., SNG, TWN, TRP).

In order for any alternative tool to compete with ultrasound for sheep PLS assessment, it would have to be either cheaper, more accurate, more convenient or able to detect PLS at earlier gestational timepoints. The metabolite panels identified in this study are more accurate, however, could they compete with the cost of ultrasound? Ultrasound tests cost between CAD$5–8 per ewe, for those producers who can access ultrasound technicians. Currently metabolite tests consisting of three or four metabolites conducted on MS instruments can be done for as little as CAD$5 per sample (excluding shipping costs). These costs can be reduced further if testing were to be optimized or more widespread. If the metabolite tests could be converted to a handheld device (such as a lateral flow assay or a simple colorimetric test) for pen side testing, then both the lower cost (perhaps as little as $3 a test) and improved convenience would make these sorts of blood tests very appealing to producers. These biomarkers have a better performance when it comes to predicting larger litter sizes in pregnant ewes. Even if we assume that these biomarkers perform comparably to ultrasound, the cost of the blood test would not vary (as it does for ultrasound scanning) based on flock size and geographical location of the farm. This would permit farms with smaller flocks and farms located in remote areas to benefit from blood-based PLS detection. If serum markers could be found effective much earlier in gestation (say at day 25 or 35) with a sensitivity or specificity that is comparable to ultrasound, then the potential of a blood test for sheep PLS would be even greater.

### Future prospects

We have shown that targeted, quantitative metabolomics technologies can be used to discover and validate serum metabolite biomarkers of sheep pregnancy and litter size. Using a large cohort of samples collected from multiple commercial flocks across Canada, we successfully identified four panels of biomarkers that can determine ewe PLS with good accuracy and precision. The performance of these markers appears to exceed that seen with ultrasound measurements within the context of this experiment. Therefore, we believe that if these biomarkers could be further optimized (for high throughput off-site assays) or translated to hand-held or pen-side tests (similar to the urine-based pregnancy detection kit for women), they could be used to routinely assess PLS in Canadian sheep flocks. We are working on developing a pen-side kit, using the panel of five biomarkers identified and validated in this study, to detect ewe pregnancy 50 days into gestation. If producers require the exact number of the litter size, a second test incorporating the two panels of biomarkers reported here could also be developed. In conclusion, translating these results for on-farm, pen-side use could significantly improve reproduction management and profitability of sheep breeding enterprises.

## Methods

All animal procedures were approved by the University of Alberta Animal Care Committee (AUP00002510) and all methods were carried out in accordance with relevant guidelines and regulations. Moreover, all methods associated with animal experiments are in accordance with the ARRIVE guidelines (https://arriveguidelines.org).

### Experimental design

The experiments were designed in two phases: (1) a discovery phase to identify candidate serum biomarkers of ewe pregnancy and litter size at the earliest timepoint in gestation, and (2) a validation phase to validate the candidate biomarkers using a sample size approximately two times larger than that used in the discovery phase.

### Discovery phase sampling

**Figure 4:**
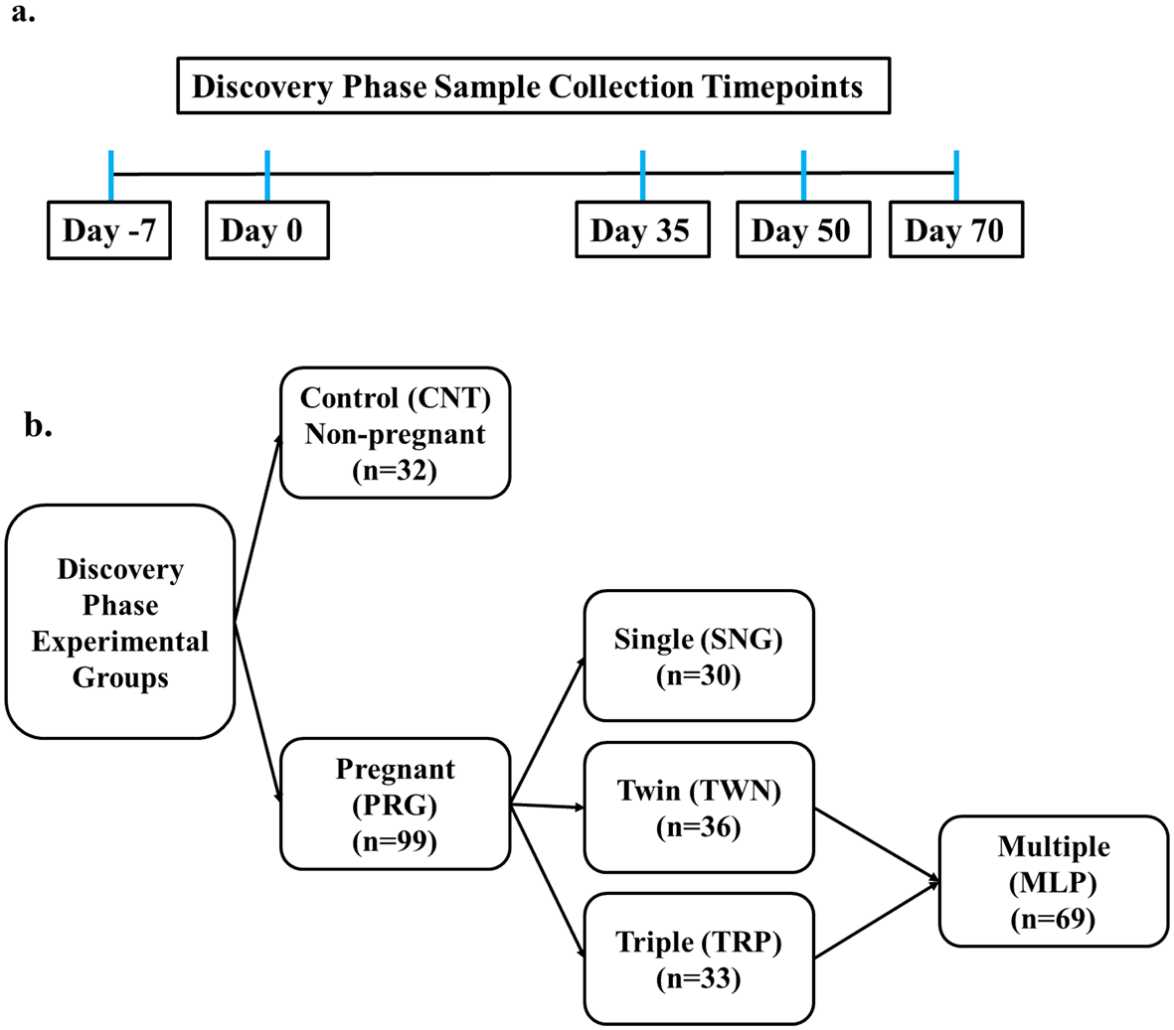
Flowchart showing experimental design and sample collection timepoints during discovery phase. (**a**) Samples were collected at five timepoints during the discovery phase; Day − 7 refers seven days prior to mating ewes and rams, Day 0 refers to the day of mating, Day 35 refers to 35 days after mating, Day 50 refers to 50 days after mating, and Day 70 refers to 70 days after mating. (**b**) Experimental groups during the discovery phase included control ewes (CNT) which were not pregnant (n = 32) and pregnant ewes (PRG) with different litter sizes (n = 99). The PRG group consisted of pregnant ewes that delivered one lamb (SNG), pregnant ewes that delivered two lambs (TWN), and pregnant ewes that delivered three or more lambs (TRP). All delivered lambs were healthy and viable.

In the discovery phase, ewes were selected from two farms (Olds College and a private farm) in Alberta, Canada, consisting of Suffolk × Dorset crosses (n = 91) and Rideau Arcott (n = 152) ewes, respectively. Blood was drawn from all animals over five timepoints throughout this phase, including seven days prior to exposing the ewes to rams (day − 7), day 0 (day of ram turnout for breeding), days 35, 50 and 70 of gestation (Fig. 4A). These animals were synchronized for estrus and the number of lambs delivered was recorded.

Based on the pregnancy outcome of all the animals included in this phase, two broad groups (Fig. 4B) were formed for statistical analyses: controls (CNT; n = 32) composed of non-pregnant, open ewes, and pregnant ewes (PRG) that delivered one or more lambs (n = 99). The CNT animals were comprised of ewes that were bred and did not deliver any lambs (n = 9) as well as the negative controls (n = 23) which were not exposed to rams. We divided the PRG animals to form three subgroups including ewes that delivered a single lamb (SNG; n = 30), ewes that delivered a twin (TWN; n = 36) and those that delivered a triplet or more (TRP; n = 33). The remaining ewes (n = 112) were not included in the analyses due to poor sample collection, missing data, and/or the producer’s decision to cull the animal.

### Animal feed

During the discovery phase, the Olds College ewes were group-housed outdoors and fed a ration of grass mix alfalfa hay with whole barley grain and a mineral supplement. Ewes at the private farm were group housed indoors in a climate-controlled barn and fed corn silage with supplemental mineral and vitamin. Initially, it was assumed all animals were pregnant with twins, and the feed rations were formulated using the SheepBytes program (https://www.sheepbytes.ca/) in compliance with National Research Council recommendations^67^. Each ewe received nutrients based on live weight of 70–75 kg (equivalent to 1.51 Mcal net energy maintenance) in early gestation.

### Estrus synchronization and breeding management

All ewes were synchronized with progesterone-bearing controlled internal drug release (CIDRs; Zoetis Canada Inc.) 14 days prior to ram turn out for breeding. To install the CIDRs, ewes were first lined in the chute and then the CIDR was inserted into the applicator by folding its wings and the tip of the applicator was gently lubricated to facilitate insertion of the device into the ewe. If the vulva appeared to be dirty, it was cleaned prior to implanting the CIDR. The applicator was then gently inserted into the vagina to release the CIDR. The applicators were disinfected between each use by dipping in a warm water and iodine solution.

Upon CIDR removal, ewes received pregnant mare serum gonadotropin (NOVORMON™, Syntex S.A., Buenos Aires, Argentina) by intramuscular injection in the rump (1 ml/ewe for the prolific Rideau Arcott breed and 2 ml/ewe for the Suffolk x Dorset crosses).

All ewes, except for the CNT group, were then grouped with the breeding rams at a ratio of no more than 10 ewes per ram. Ram turnout at the Alberta private farm location occurred on November 4th, 2017, with ewes lambing between March 29th and April 5th, 2018. Ram turnout at the Olds College location occurred on October 4th and 11th, 2017 (groups A and B, respectively), with ewes lambing between February 26th and March 28th, 2018. Lambing at each location was observed and recorded by farm staff.

### Laparoscopic reproductive examination

A subset of the negative controls was examined at day 50 of gestation using laparoscopy to visually observe and approve ovarian health. Animals were restrained using a cradle and anesthetized by intravenous injection of a combined sedative of 0.6 mg/mL xylazine (Vetoquinol Canada Inc., ON, Canada) and 2 mg/mL Ketamine (Vetoquinol Canada Inc., ON, Canada). Once on the cradle, the anesthetized ewe was lifted from its rear, bringing the back two legs up while the head and front two legs are down. Approximately six inches from each teat was clipped and cleaned with a 4% chlorhexidine scrub (Ceva Animal Health Inc., ON, Canada) and 99% isopropyl alcohol. The clipped areas provided a point of entry for the scope on one side and a cannula on the other. A moderate amount of CO_2_ was introduced into the abdominal cavity through a trocar going into one of the clipped points. The laparoscope was introduced into the cannula to see the ovaries. The ovaries of all open ewes were observed and approved by a veterinarian as reproductively sound and not showing any apparent abnormalities. The cannulas were then removed and the skin was stapled to close the two holes. The animals were gently rolled off the cradle and within five minutes they were relieved from the anesthesia. All utensils were maintained and cleaned in a dilute iodine solution (West Penetone Inc., QC, Canada) between each animal examination.

### Ultrasound diagnosis

All bred ewes were trans-abdominally scanned (Sonosite M-Turbo ultrasound machine, FUJIFILM Sonosite Inc., ON, Canada) for pregnancy and litter detection while standing in a chute at day 50 of gestation by an experienced technician for each province. Certified technicians reported pregnancy as open (no detectable fetus present), single (detection of only one fetus), twins (detection of two fetuses), and triplets or more (detection of more than two fetuses). All ultrasound assessments were reconciled with the actual lambing records from each flock.

### Validation phase sampling and feeding

During the validation phase, ewes were selected from two farms in Alberta (Suffolk and Canadian Arcott crosses at Lakeland College [n = 65], and Suffolk crosses at a private farm [n = 12]) and two farms in Ontario (Rideau Arcotts and Suffolk crossed with Rideau Arcott at private farm one [n = 55], and Dorset and Rideau Arcott crosses at private farm two [n = 111]). Each farm used “typical” Canadian feed rations. In particular, sheep were fed either (1) grass-legume hay mixtures with grain (barley or corn) or (2) corn silage or haylage. Specifically, one farm in Ontario and one farm in Alberta fed silage/haylage, whereas one farm in Alberta and one farm in Ontario fed the hay and grain mix. Based on the discovery phase results, blood was only drawn from all animals at a single timepoint (day 50 of gestation). All ewes were naturally mated to the rams at a ratio of 10:1, none of which were synchronized for estrus. All ewes had their lambing outcome recorded and categorized similar to the discovery phase (i.e., CNT, PRG, SNG, TWN and TRP).

### Blood collection and processing

Blood samples from all ewes of both phases (discovery and validation) were drawn from the jugular vein. Samples were collected using 21-gauge needles (PrecisionGlide^®^, USA) and vacutainers coated with no anticoagulant (BD Vacutainer, USA) for a maximum volume of 10 mL. Blood samples were kept on ice upon collection for a maximum of 30 min. Samples were then centrifuged (Beckman Coulter, USA) for 30 min at 17,700 rpm at 4 °C. The supernatant serum was then transferred to Eppendorf tubes (Axygen, USA) and snap frozen using liquid nitrogen. Frozen serum samples were labelled and stored at − 80 °C until used for metabolomic analyses.

### Metabolomics experiments

All ewe serum samples were analyzed using nuclear magnetic resonance (NMR) spectroscopy and liquid chromatography tandem mass spectrometry (LC–MS/MS). A thorough description of sample preparation and analysis methods for each platform is provided in Goldansaz et al.^23^. In brief, for the NMR analysis, all serum samples were filtered using a 3 kDa ultrafiltration device to remove the macromolecules (i.e., proteins and lipoproteins). A total sample volume of 250 µL (including the serum and buffer solution) was introduced to a 700 MHz Avance III (Bruker, USA) spectrometer equipped with a 5 mm HCN Z-gradient pulsed-field gradient cryoprobe. The 1D ^1^H-NMR spectra were then collected, processed and analyzed using methods previously described and a modified version of the Bayesil automated NMR analysis software package^68^. For the LC–MS/MS metabolomic analysis, serum samples were analyzed using an in-house quantitative metabolomics kit (called TMIC Prime) run on an Agilent 1260 series UHPLC system (Agilent Technologies, Palo Alto, CA) coupled with an AB SCIEX QTRAP^®^ 4000 mass spectrometer (Sciex Canada, Concord, Canada). A detailed description of the methods, kit design, workflow and data analysis is given in Goldansaz et al.^23^.

### Statistical analyses

To conduct a standard categorical analysis and identify the relevant serum PLS biomarkers, we categorized the animals into six different groups based on their pregnancy outcome (i.e., CNT, PRG, SNG, TWN, TRP, MLP). Metabolomic datasets from the two platforms were pre-processed and normalized using standard methods available via MetaboAnalyst 4.0^69^. Metabolites that had > 20% missing values were removed from the dataset prior to statistical analyses. Univariate and multivariate statistical analyses, including fold change, student’s t-test, volcano plot analysis, and partial least squares discriminant analysis (PLS-DA) were conducted using MetaboAnalyst. The PLS-DA plot helped visualize the separation of each animal group based on their corresponding serum metabolome, and its significance was verified using permutation testing (n = 1000). The PLS-DA analyses that were significant were also evaluated for the top 15 VIP features, revealing those metabolites that had the most significant contribution to separating the comparison groups. Biomarkers were identified and evaluated using the biomarker module in MetaboAnalyst 4.0^69^. This module automatically selects subsets of statistically significant metabolites (initially identified via PLS-DA analysis and validated by permutation analysis using n = 1000 and p < 0.05). This module then performs a series of logistic regression calculations on the normalized and scaled metabolite concentration values and calculates the ROC curves as well as the AU-ROC values to identify the optimal set of biomarkers. Individual or multiple metabolite profiles with an AU-ROC ≥ 0.70 were considered as candidate biomarkers for each trait. The threshold for statistical significance reported in this manuscript is a p-value < 0.05 and a Benjamini–Hochberg false discovery rate (or Q-value) < 0.05, unless otherwise mentioned. Also, a 0.05 < p-value < 0.10 is referred to as a tendency while, differences with a p-value > 0.10 are referred to as not significant.

## Supplementary Information

Supplementary Information.

## Data Availability

All data are publicly available online at the Livestock Metabolome Database (www.lmdb.ca).
