# Supplementary material for: Predictive blood biomarkers of sheep pregnancy and litter size
Source: Sci Rep. 2022 Jun 20;12:10307. doi: 10.1038/s41598-022-14141-w (PMC9209467; doi:10.1038/s41598-022-14141-w)

## Supplementary Material

**Tables**

**Table 1. Average concentration (and standard deviation) of significantly changed metabolites identified at day 50 of the discovery phase.**

| Day 50 Metabolites | Concentration (µM) | Standard Deviation |
| --- | --- | --- |
| Acetic acid | 513.91 | 313.87 |
| Urea | 2034.64 | 765.61 |
| SM (OH) C24:1 | 0.90 | 0.39 |
| lysoPC a C26:0 | 0.11 | 0.05 |
| lysoPC a C26:1 | 0.07 | 0.03 |
| Tryptophan | 41.87 | 13.86 |
| C3 (propionylcarnitine) | 0.37 | 0.14 |
| Carnosine | 31.96 | 8.37 |
| Alpha-aminoadipic acid | 1.49 | 0.54 |
| Putrescine | 0.03 | 0.02 |
| Trimethylamine N-oxide | 7.62 | 10.16 |
| lysoPC a C18:2 | 15.97 | 7.31 |
| Hippuric acid | 24.11 | 12.78 |
| lysoPC a C14:0 | 1.18 | 0.43 |
| L-arginine | 210.50 | 90.99 |
| lysoPC a C16:1 | 2.14 | 0.91 |
| L-carnitine | 36.91 | 11.29 |
| Methionine | 31.22 | 9.66 |
| Valine | 223.59 | 78.70 |
| L-lactic acid | 2880.60 | 1671.87 |
| Isobutyric acid | 13.27 | 5.28 |
| Methionine-sulfoxide | 4.57 | 2.76 |
| Spermidine | 0.05 | 0.02 |
| Acetyl-ornithine | 7.07 | 3.61 |
| Kynurenine | 3.93 | 2.02 |
| SM C20:2 | 0.26 | 0.12 |
| 3-hydroxybutyric acid | 390.29 | 190.49 |
| Dimethyl sulfone | 67.49 | 55.16 |
| L-lysine | 182.44 | 76.72 |
| L-ornithine | 136.39 | 67.32 |
| C0 (Carnitine) | 45.08 | 14.61 |
| Methanol | 47.80 | 45.12 |
| D-glucose | 3805.51 | 1171.42 |
| Tyrosine | 66.54 | 27.27 |

**Figures**

**Figure 1. Boxplot of biomarkers of sheep pregnancy (CNT vs PRG).** Boxplot comparing the normalized concentration of the biomarkers of sheep pregnancy (methanol, L-carnitine, D-glucose, L-arginine, urea).


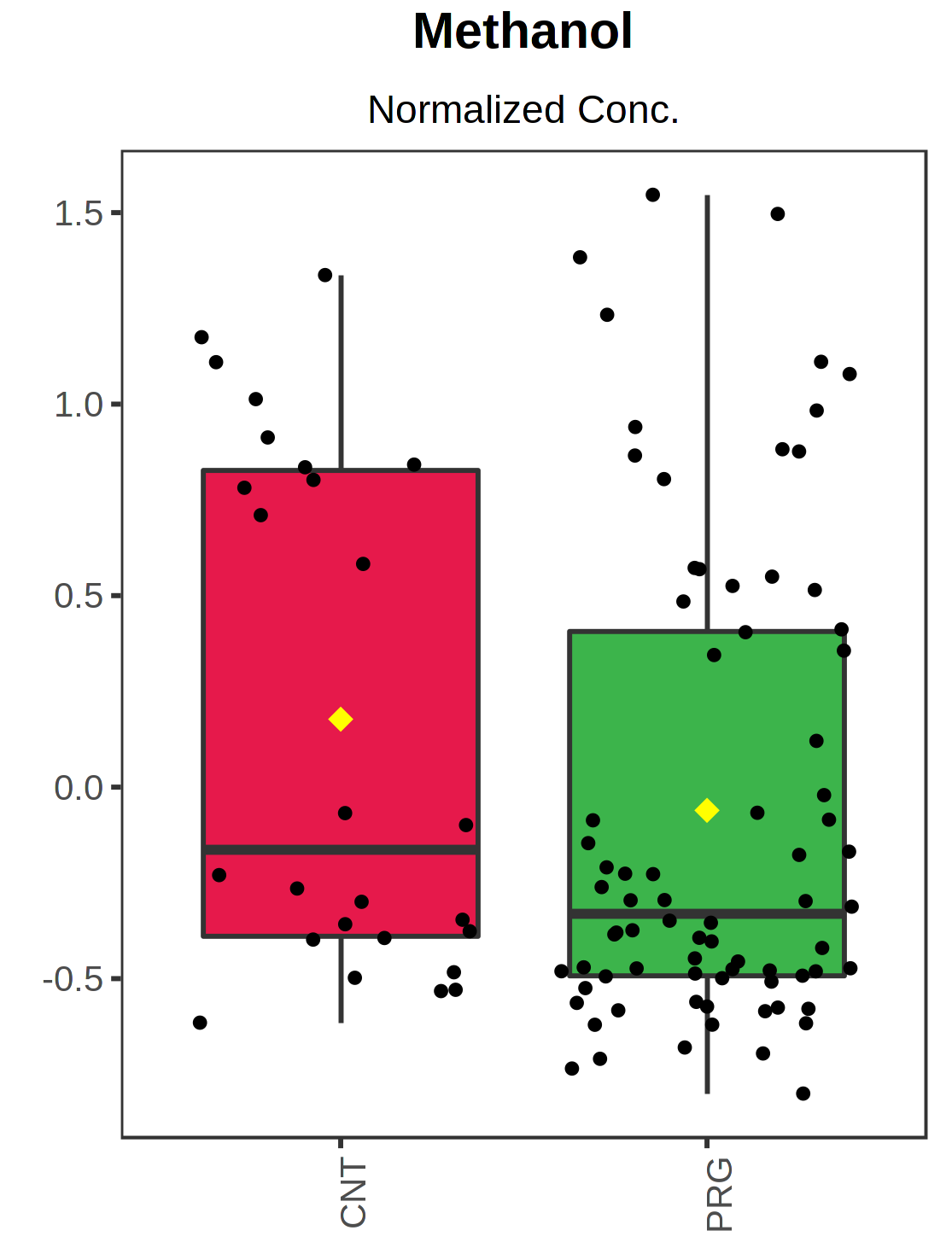


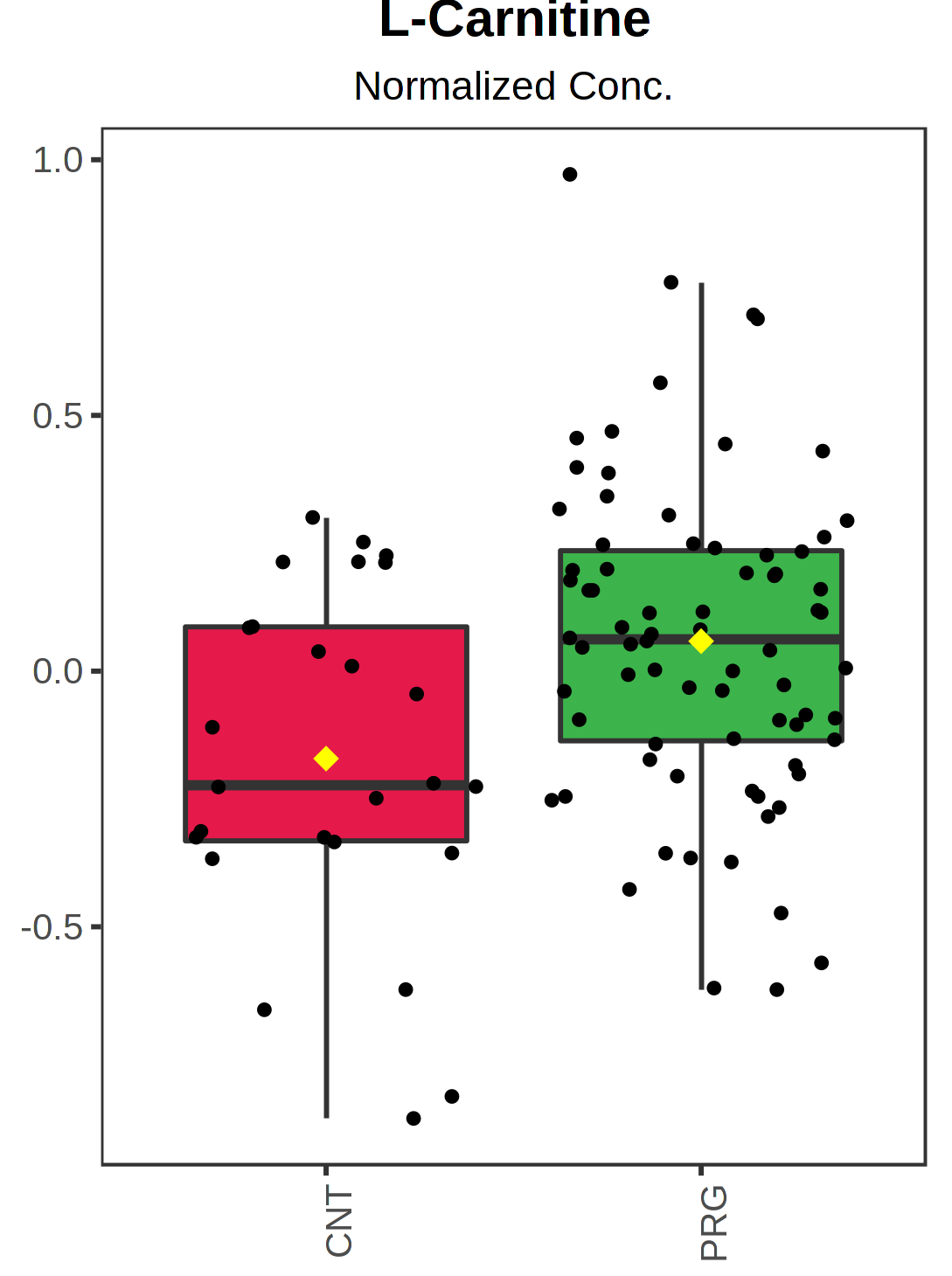


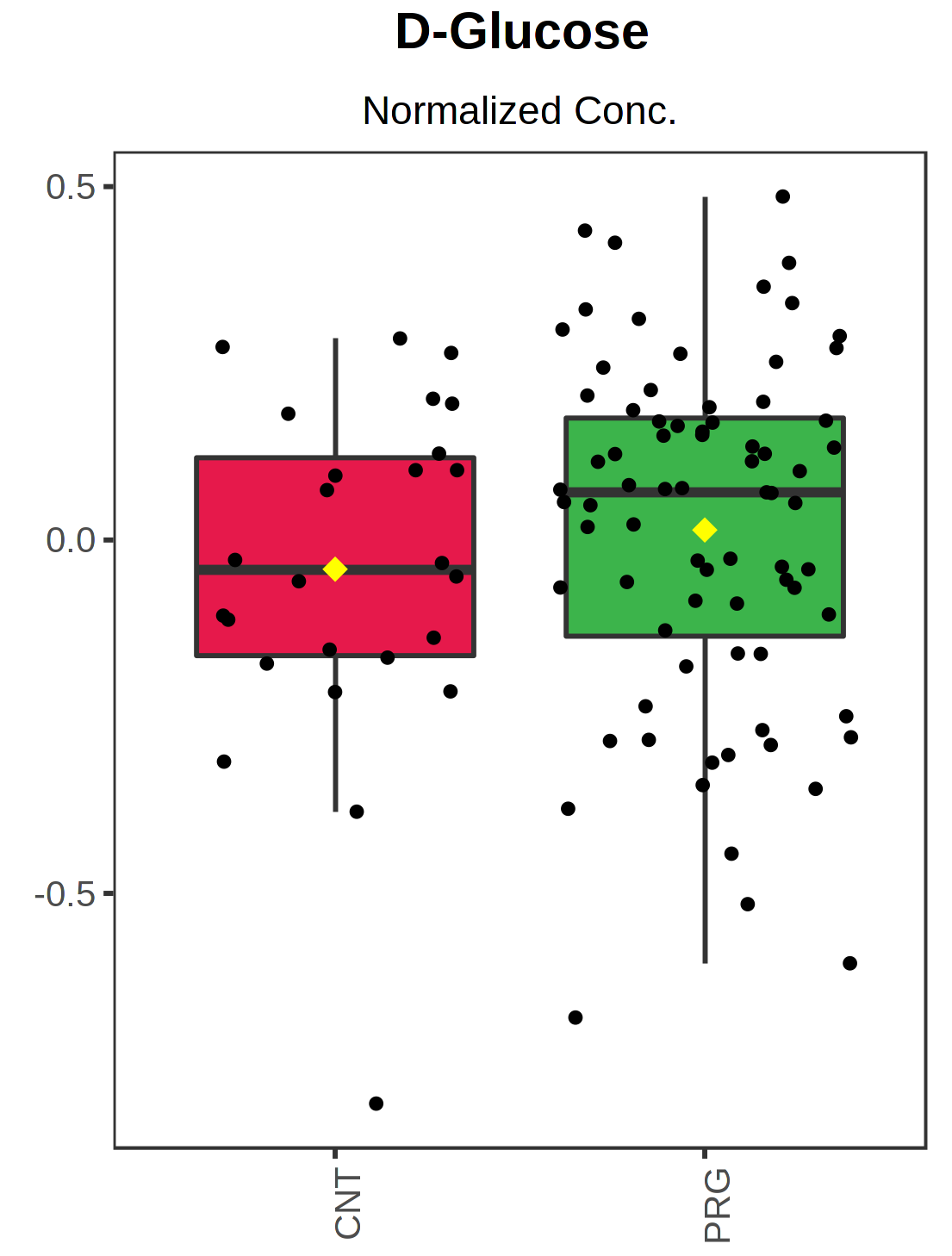


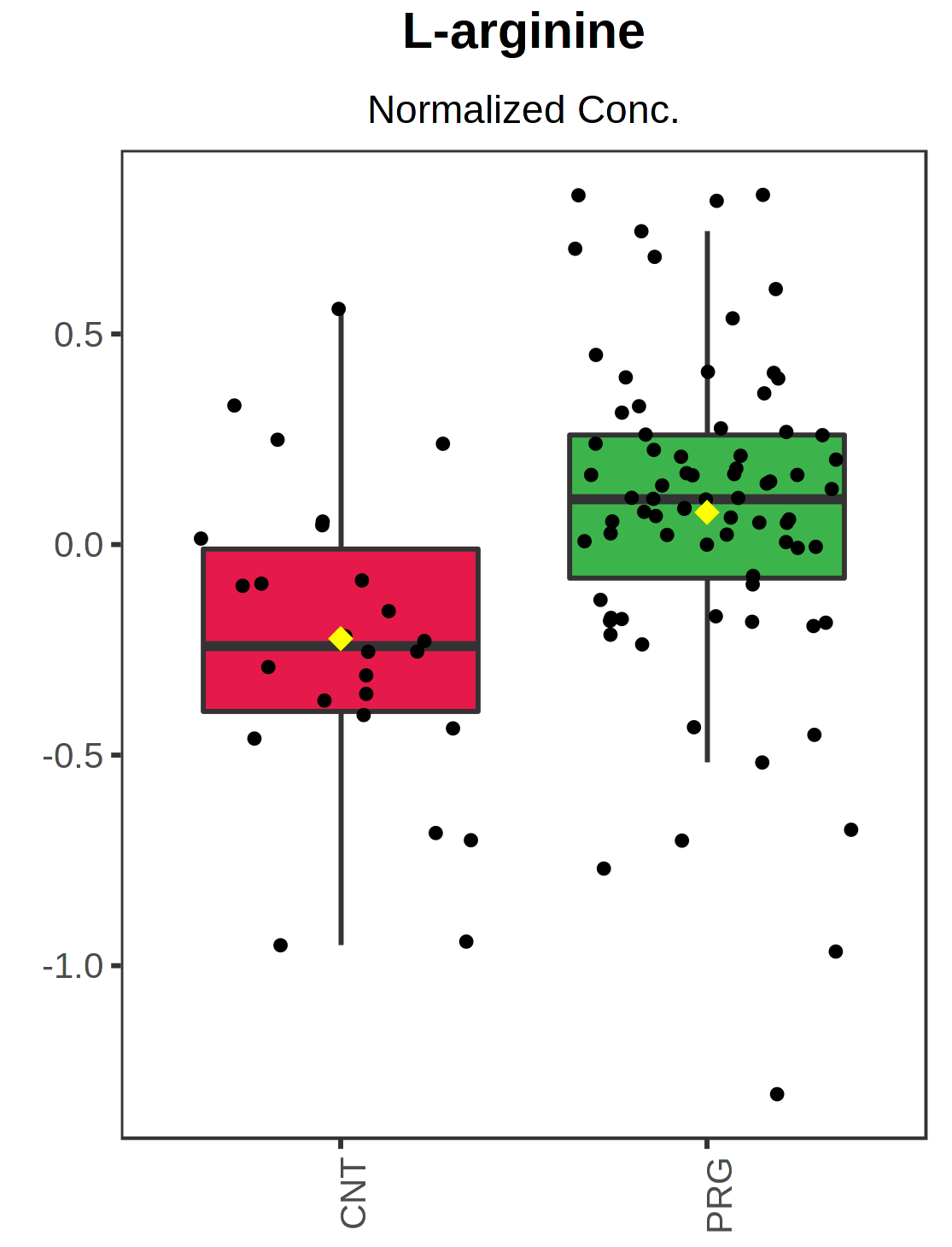


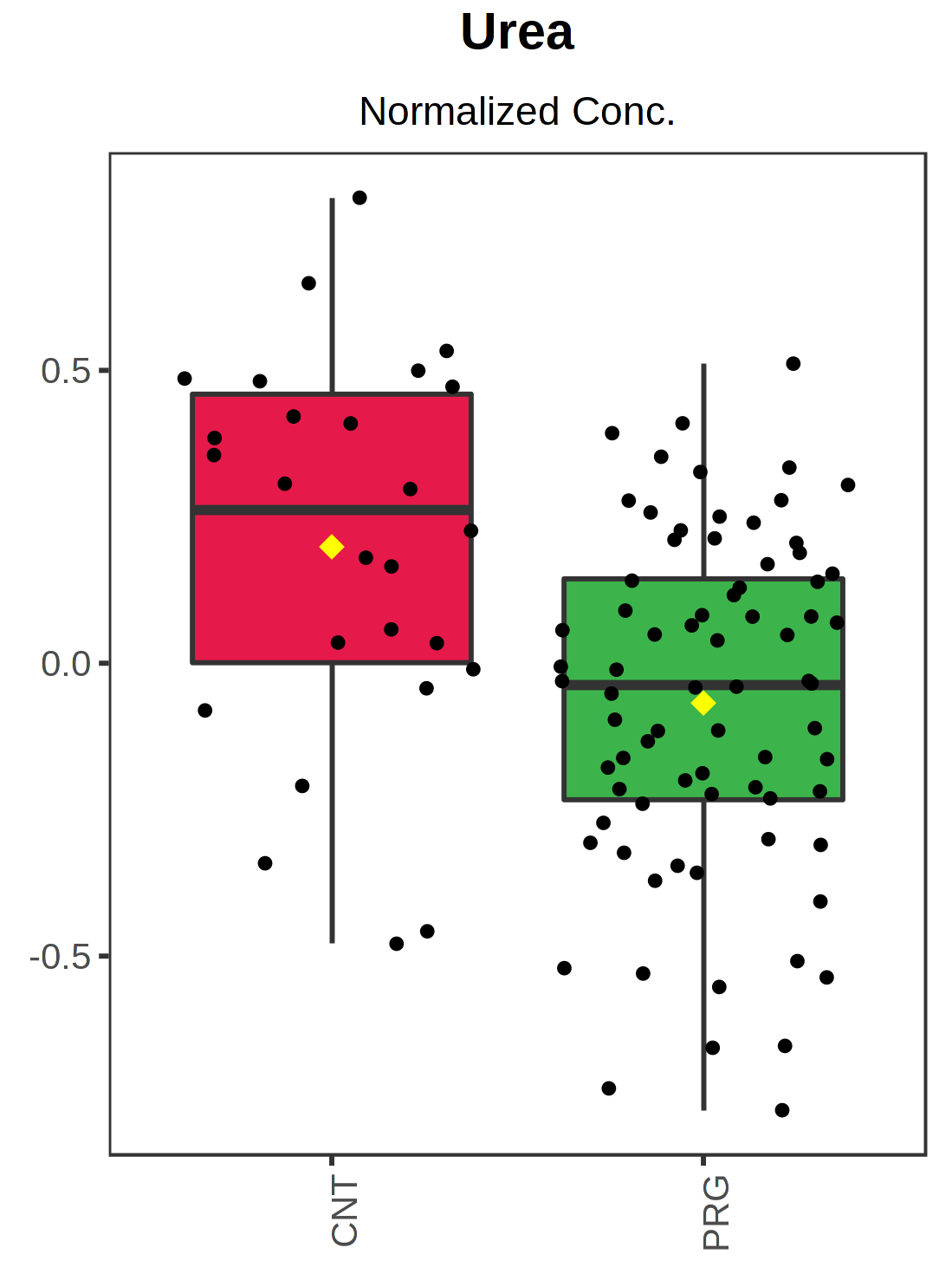


**Figure 2. Boxplot of biomarkers of sheep litter size (****SNG vs TRP).** Boxplot comparing the normalized concentration of the biomarkers of sheep litter size based on comparison between SNG and TRP groups (methionine, L-carnitine).


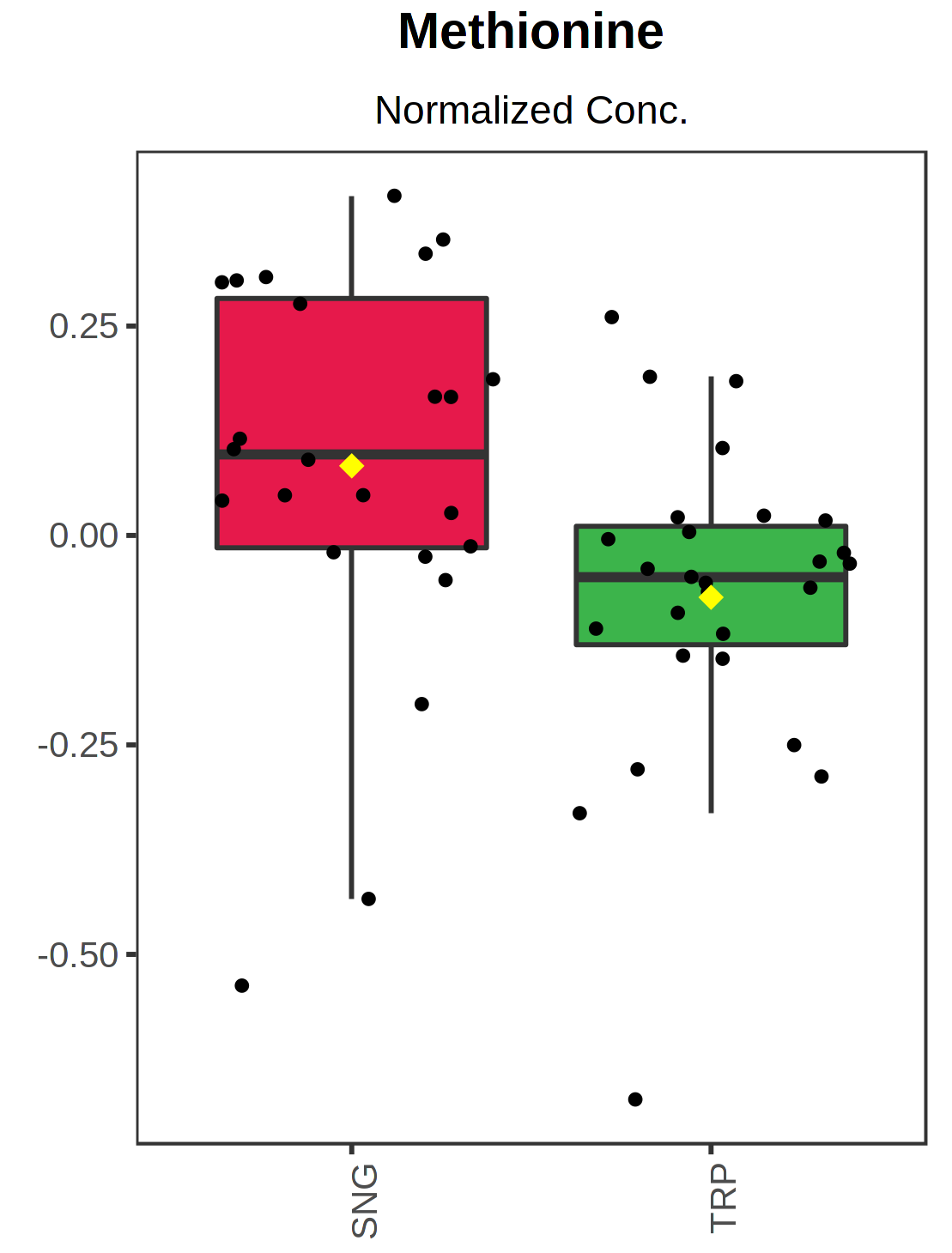


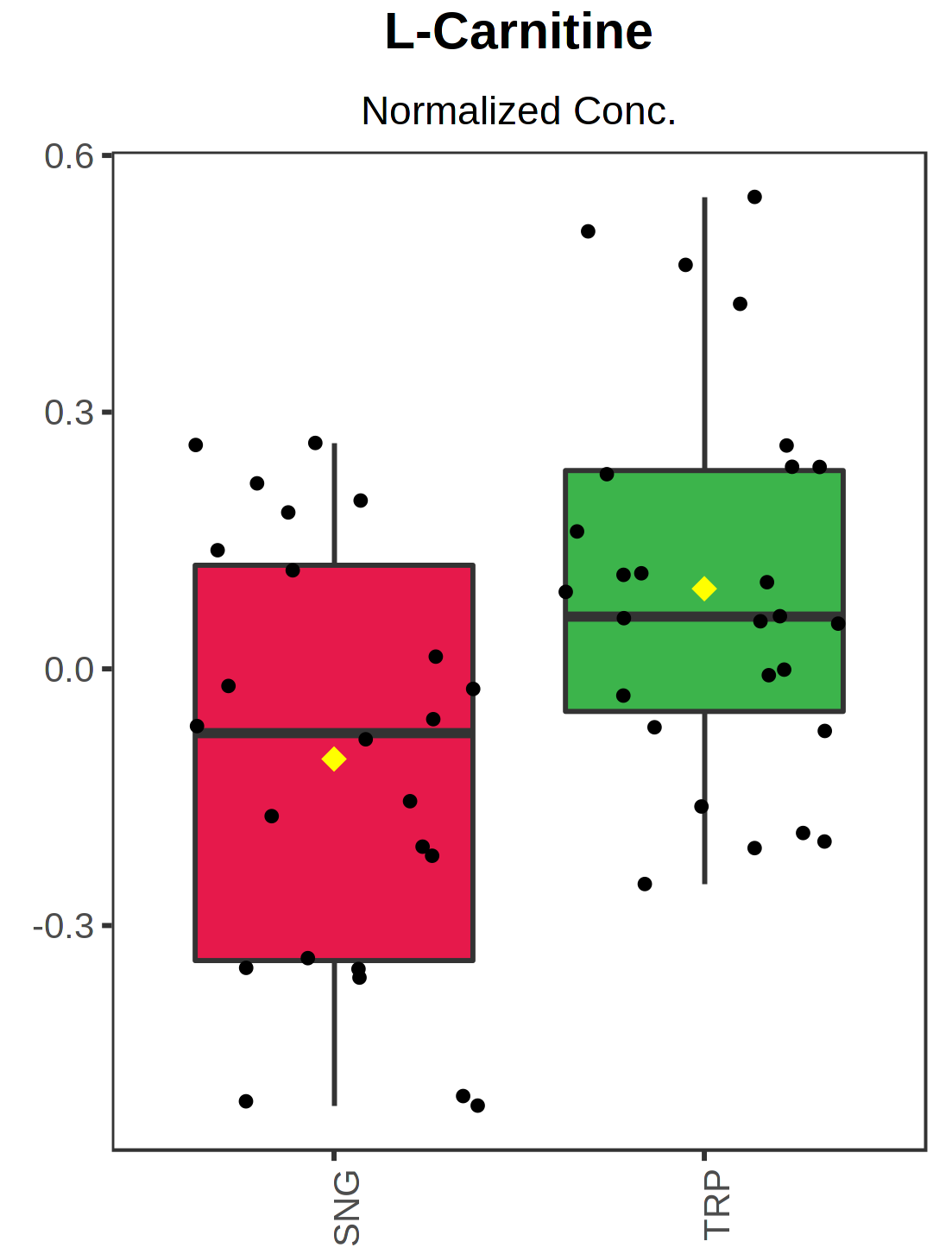


**Figure 3. Boxplot of biomarkers of sheep litter size (TWN vs TRP).** Boxplot comparing the normalized concentration of the biomarkers of sheep litter size based on comparison between TWN and TRP groups (isobutyric acid, L-lactic acid, L-carnitine, valine, tyrosine, methanol).


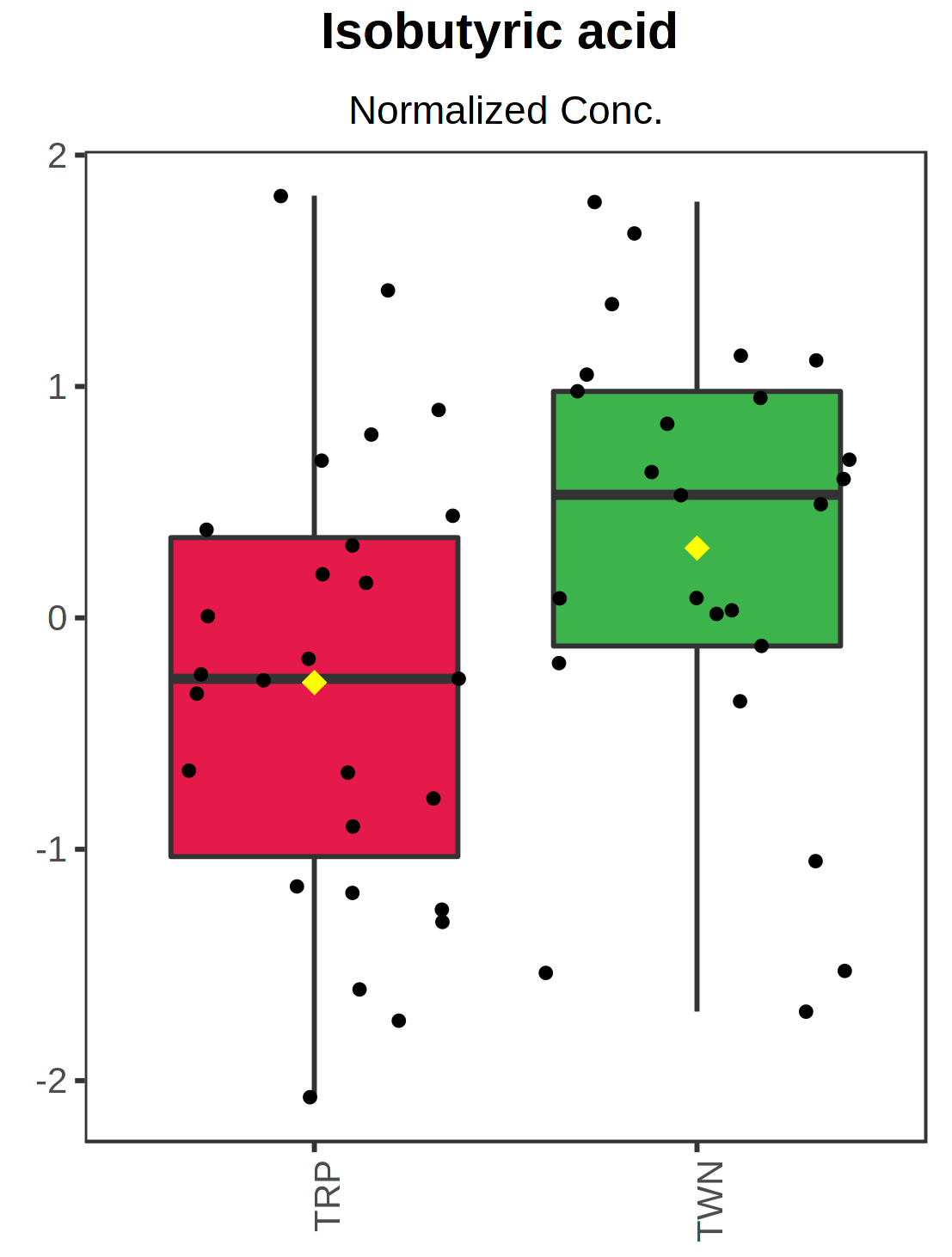


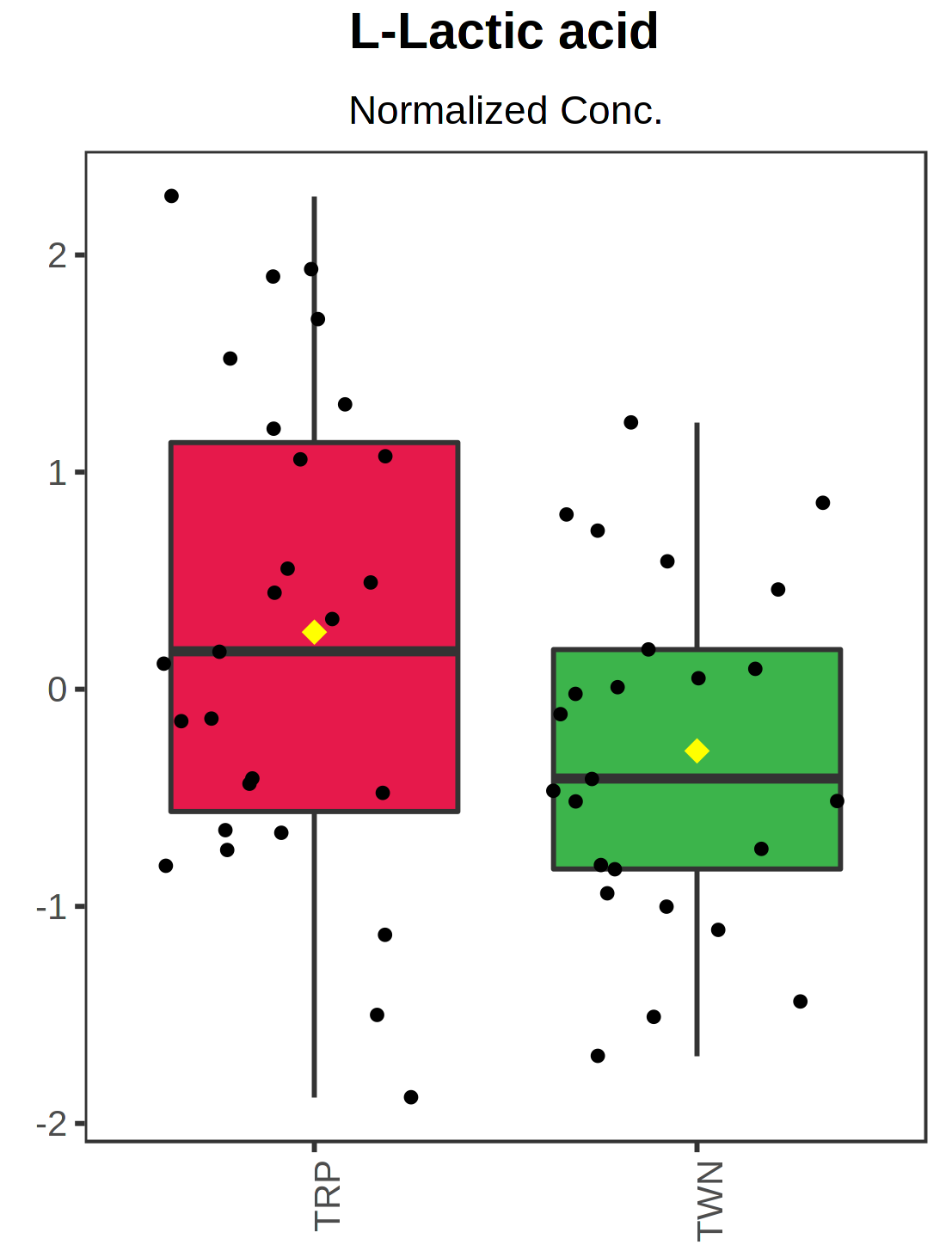


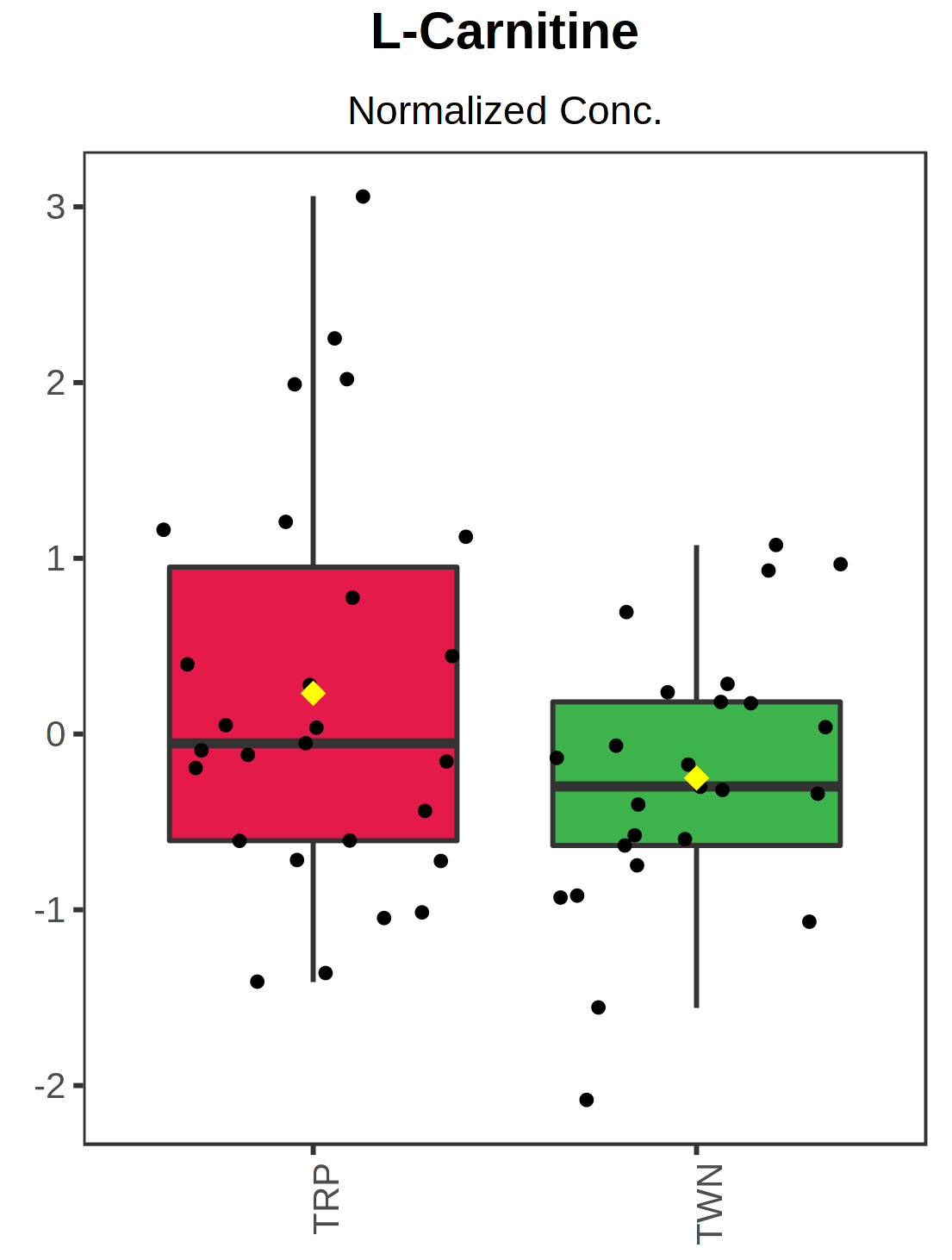


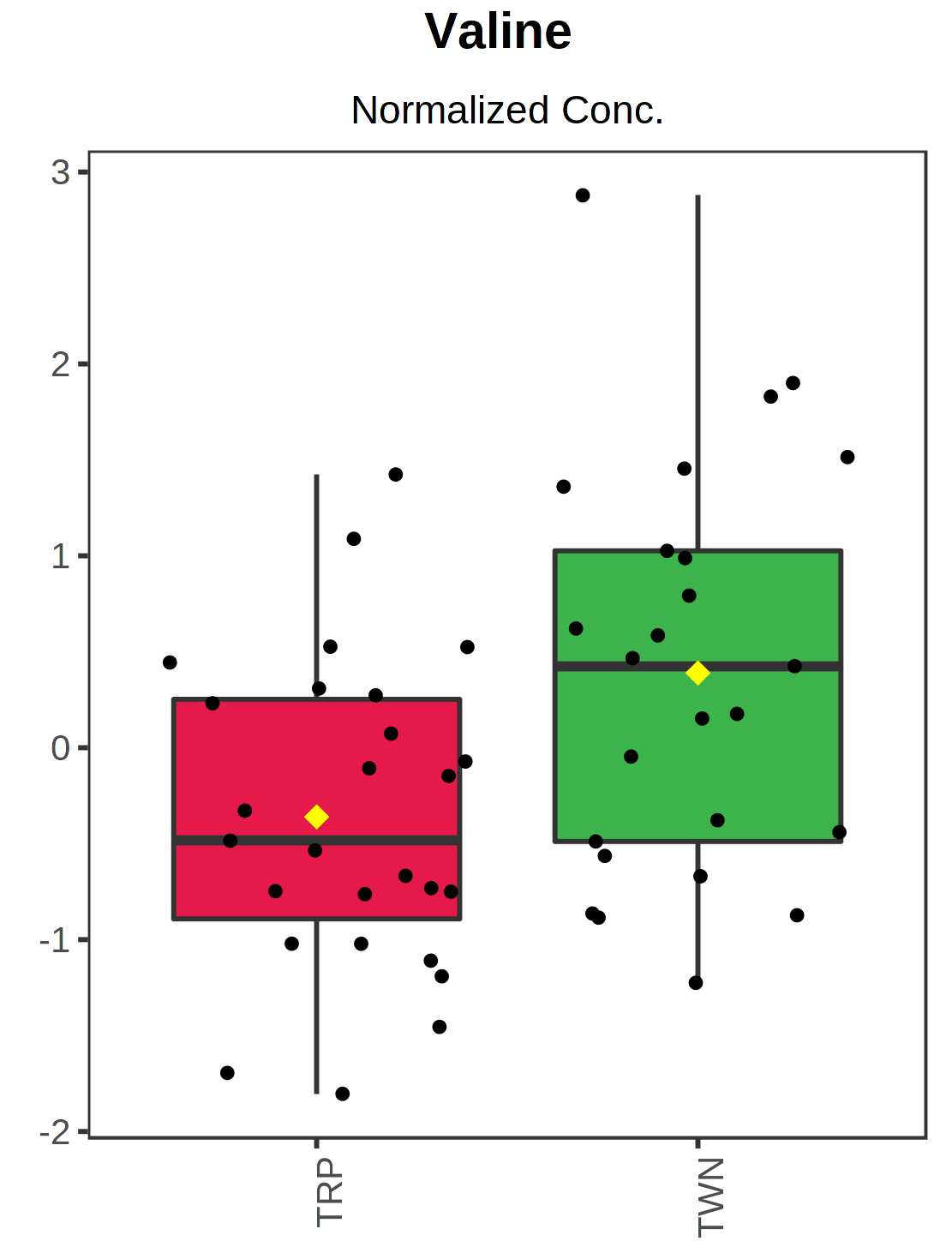


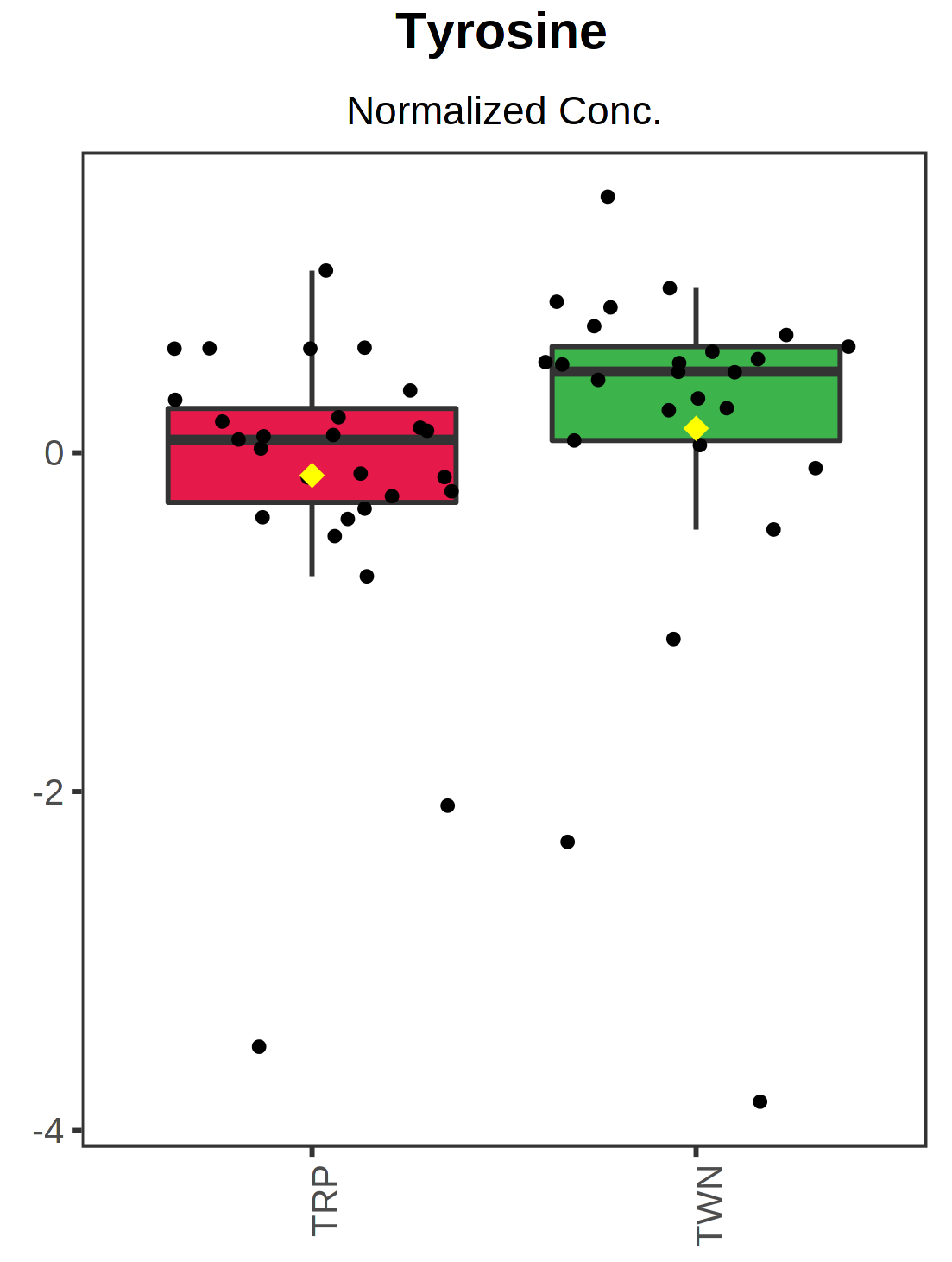


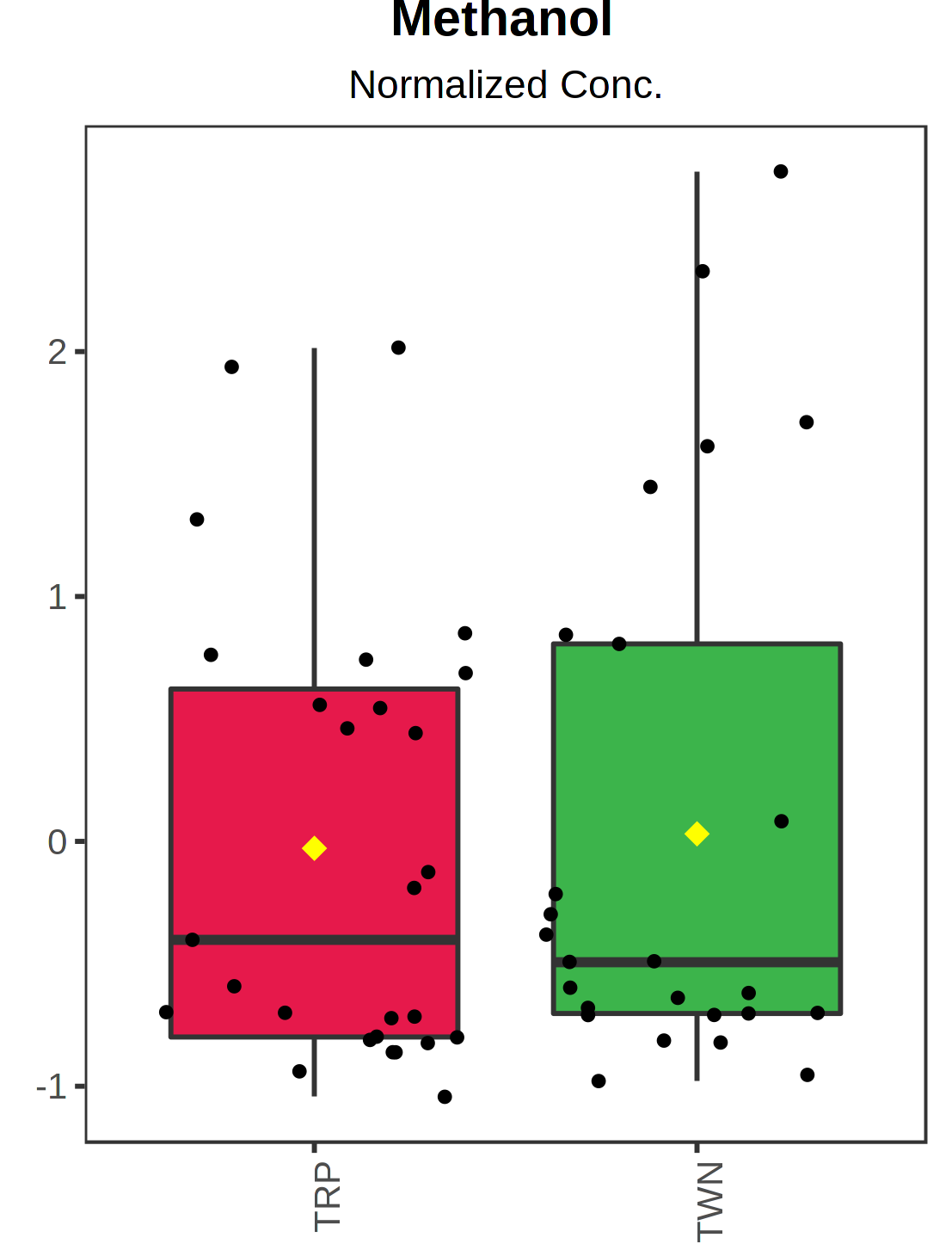

Supplement: Supplementary file 1 — Supplementary Information. [file 41598_2022_14141_MOESM1_ESM.docx]
